# Comparative studies targeting the best dyeing conditions for pure, blend and modified fabrics with novel nano-disperse reactive dyes and their biological activity

**DOI:** 10.1038/s41598-026-55597-4

**Published:** 2026-06-04

**Authors:** Hala F. Rizk, Hamada M. Mashaly, Hamada S. A. Mandour, Khalil M. Saad-Allah, Mohamed A. B. Mohamed, Mohamed R. Sadek, Amira K. Fares

**Affiliations:** 1 Department of Chemistry, Faculty of Science, Tanta University, Tanta, 31527 Egypt; 2 Dyeing and Printing Department, Textile Research and Technology Institute, National Research Center, Giza, 12622 Egypt; 3 Department of Botany, Faculty of Science, Tanta University, Tanta, 31527 Egypt; 4 Alsalam University, Kafr El-Zayat, Egypt

**Keywords:** Disperse reactive, Vinyl sulfone, Cyanuric chloride, Dyeing primary parameter, EWG and EDG, Antimicrobial activity, Biotechnology, Chemistry, Materials science, Nanoscience and technology

## Abstract

Here, in the current work, three different types of novel nano-disperse reactive dyes (**3a**, **3b**, and **4**) based on the pyrazolone moiety with one or two reactive centers were synthesized and used to study the effect of the kind and number of reactive groups on dyeing performance. The structure of the synthesized nano-disperse dyes was confirmed using different spectroscopic techniques. The dyeing properties of newly synthesized dyes have been investigated for dyeing different fabrics under traditional conditions. On the other hand, we studied the effect of changes in pH, dyeing bath temperature, and dyeing time through statistically designed experiments to determine the optimal conditions for achieving high fixation and good color fastness. After careful investigation, we found that dye **3a**, which has a vinyl sulfone group as a reactive group center in its structure, gave the best and highest value of fixation (K/S%) ranging from (12.17–50.85) in almost all of the examined fabrics (polyester, cotton, polyester/wool, polyester/cotton and polyester/chitosan) except wool, taking into consideration the change of suitable temperature, pH level, and dyeing time for each type of fabric. Critically, the synthesized dyes exhibited significant and structure-dependent antimicrobial activity against the Gram-positive (*S. epidermidis*, *S. haemolyticus*, MRSA) and Gram-negative (*E. coli*, *K. pneumoniae*, *P. aeruginosa*) bacteria and the pathogenic yeast *C. albicans*. Data revealed that dye **3b** possessed exceptional broad-spectrum potency, specifically against *S. haemolyticus*, MRSA, *K. pneumoniae*, and *C. albicans*.

**Supplementary Information:**

The online version contains supplementary material available at 10.1038/s41598-026-55597-4.

## Introduction

Disperse reactive dyes are widely used to color a wide range of textiles, including pure, blended, and modified fabrics^1–3^. These dye systems’ exceptional fastness and enough fabric coverage provide advantages by successfully resolving the leveling problems that come with various fabric types^3^. Moreover, attempts have been undertaken to create substitute disperse dyes that have vinyl sulfone and/or chlorotriazine reactive groups^4,5^. When the dyes with sulphatoethylsulphone were applied at 90 °C and a pH range of 5–12, they showed exceptional binding rates and wash fastness on the application of these dyes on various fabrics^3,6,7^. Although vinyl sulfone (VS) is classified as a monofunctional reactive group, it can be converted into a hetero bifunctional reactive group by adding chlorotriazine to dye structures. The vinyl sulfone reactive group participates in a nucleophilic addition reaction with cotton fabric in alkaline circumstances conditions, whereas the chlorotriazine/vinyl sulfone (CT/VS) reactive group undergoes a nucleophilic substitution reaction^8–12^. The triazinyl group helps to increase substantivity, usually between 30 and 60%, and makes exhaust coloring easier; these dyes were mainly created for this purpose^13^. Many variables, such as reactivity, diffusibility, substantivity, and the position of the reactive groups, greatly influence the dye’s adhesion efficiency to cellulosic fabrics. By combining two reactive groups with different levels of reactivity (CT at 80 °C; VS at 50 °C), the range of application temperatures is expanded^14^. Whether dyeing conditions, which differ greatly depending on the kind of cloth, depend on whether the fiber is synthetic or natural (made from cellulose or protein). For example, disperse dyes are used to color polyester at temperatures over 100 °C and pH levels between 4.5 and 5.5 for 30 to 60 min. Traditional temperature, pH level, time, and dye type are crucial parameters for getting vibrant, long-lasting colors^3,15,16^. Conversely, cotton is dyed with reactive dyes at temperatures between 40 and 60 °C and pH levels greater than 8 for at least 60 min. Meanwhile, wool is exposed to acid dyes for 60 to 90 min at pH levels between 4 and 6 and temperatures between 90 and 100 °C. As previously mentioned, the dyeing of blended fabrics, such as polyester/cotton, polyester/wool, and polyester/modified dyeing, is achieved by using different dyes in a single dye bath under various conditions^17–19^. In this study, we attempted to evaluate how electron-donating groups (EDG) and/or electron-withdrawing groups (EWG) influence the colour properties of our new synthetic disperse reactive dyes. Furthermore, we will evaluate the impacts of a variety of variables on the final dyeing qualities, such as temperature, pH, and dyeing duration, to determine the optimal conditions that can be applied for the dyeing process to provide the highest substantivity of dyes on the tested fabrics. Our research will help design more efficient dyeing methods by shedding light on the connection between chemical structure and dye performance.

A particularly compelling direction is the development of textile dyes with intrinsic antimicrobial properties. This addresses a growing demand for hygienic and protective textiles in medical, sportswear, and domestic applications^20^. Nano-structuring dye particles *via* methods like ball milling can enhance this bioactivity by increasing surface area and interaction with microbial cells^21^. The pyrazolone moiety, a heterocyclic scaffold prevalent in pharmaceutical chemistry, is recognized for its diverse biological activities, including antimicrobial effects^22,23^. Recent studies have further demonstrated that pyrazole-based compounds exhibit significant activity against drug-resistant bacteria and fungi, often through mechanisms involving membrane disruption and inhibition of biofilm formation^24,25^. However, most of these reports focus on free antimicrobial agents rather than on covalently fabric-bound dyes, leaving a critical gap; it remains unclear whether the antimicrobial potency of pyrazolone derivatives is retained after their integration into disperse reactive dye structures and subsequent fixation onto textiles. Bridging this gap is essential because a dye that performs well in solution may lose activity once anchored to a fabric surface due to steric hindrance, altered electronic distribution, or reduced accessibility to microbial targets. Without this knowledge, the design of multifunctional textiles risks pursuing structures that are ineffective in real-use conditions. Therefore, this study explicitly bridges that gap by systematically comparing three novel nano-disperse reactive dyes based on a pyrazolone core, varying in electronic substituents (NO_2_ vs. NH_2_) and number of reactive groups (mono- vs. bi-functional), to assess both their dyeing performance and, crucially, their retained antimicrobial activity against a panel of multidrug-resistant clinical isolates. Unlike previous works, we provide a direct structure-activity relationship that correlates molecular design (electron-donating/withdrawing groups, reactive center count) with dual functionality: color fixation and biocidal efficacy. This comparative assessment reveals, for the first time, that an electron-donating group enhances broad-spectrum activity while a second reactive group for textile anchoring may compromise antimicrobial potency, a finding with direct implications for future multifunctional dye engineering.

## Experimental

### Chemistry

The supplementary file contained spectral data and elemental analysis. All of the materials were dyed using Ahiba IR laboratory dyeing equipment. The degree of fatigue in dyed fabrics was evaluated using spectrophotometry. The materials used for dyeing were commercially available 100% polyester with a surface mass of 109 g/m², 100% cotton with a surface mass of 117 g/m², 100% wool with a surface mass of 264 g/m², a polyester/cotton blend (50/50) with a surface mass of 117 g/m², and a polyester/wool blend (55/45) with a surface mass of 177 g/m². The published technique^26^ was followed in the preparation of the polyester modified with chitosan.

#### General procedure for synthesis of compounds **3a**, **3b**

A solution of 2-((4-aminophenyl)sulfonyl)ethyl hydrogen sulfate (2.81 g, 0.01 mol) dissolved in strong (concentrated) hydrochloric acid was gradually mixed with a solution of sodium nitrite (0.69 g, 0.01 mol). For an hour, the resultant mixture was constantly stirred while being kept between 0 °C and 5 °C. Furthermore, a cooled solution comprising 3-methyl-1-aryl-1*H*-pyrazol-5(4*H*)-one (**2a**,** 2b**) (0.01 mol) in ethanol (20 ml) and sodium acetate (2.5 g) was mixed with the diazonium salt dropwise while stirring. At 0–5 °C, the mixture was additionally agitated for an additional hour. After that, the solid product was filtered, washed with cold water, and finally dried.

##### Synthesis of 2-((4-(2-(3-methyl-1-(4-nitrophenyl)-5-oxo-1,5-dihydro-4 H-pyrazol-4-ylidene)hydrazineyl)phenyl)sulfonyl)ethyl hydrogen sulfate (**3a**)

Yellow powder, yield: 91% (4.66 g), m.p: 140–142 °C. IR (KBr, cm^− 1^) 3481 (OH), 3117 (NH), 3086 (Ar-H), 2895 (Aliph-H), 1562 (C = O), 1517 (C = N), 1501 (C = C), 1320 (NO_2_), 1129 (S = O). ^1^H NMR (500 MHz, DMSO-*d*_*6*_) *δ* 2.19 (s, 3 H, CH_3_), 3.65 (t, 2 H, CH_2_), 3.73 (t, 2 H, CH_2_), 5.42 (s, 1H, OH exchangeable by D_2_O), 7.57–8.22 (m, 8 H, CH-Ar), 13.21 (s, 1H, NH exchangeable by D_2_O). ^13^C NMR (101 MHz, DMSO-*d*_*6*_) 12.8 (CH_3_), 55.0 (CH_2_), 57.8 (CH_2_), 116.9-150.5 (C-aromatic), 127.6 (*C* = N-NH), 151.0 (C = N of pyrazolone), 156.4 (C = O). Anal.Calc for C_18_H_16_N_6_O_11_S_2_ (511.42): Calcd: C: 42.27; H: 3.35; N: 13.69; S: 12.54. Found: C: 42.21; H: 3.28; N: 13.73; S: 12.59, MS (*m/z*): 511.32 [M^·+^].

##### Synthesis of 2-((4-(2-(1-(4-aminophenyl)-3-methyl-5-oxo-1,5-dihydro-4 H-pyrazol-4-ylidene)hydrazineyl)phenyl)sulfonyl)ethyl hydrogen sulfate (**3b**)

Yellow powder, yield: 98% (4.76 g), m.p: 178–180 °C. IR (KBr, cm^− 1^) 3470 (OH), 3230 (NH), 3048 (Ar-H), 2914 (Aliph-H), 1591 (C = O), 1463 (C = N), 1442 (C = C), 1135 (S = O). ^1^H NMR (500 MHz, DMSO-*d*_*6*_) *δ* 2.20 (s, 3 H, CH_3_), 3.39 (t, 2 H, CH_2_), 3.82 (t, 2 H, CH_2_), 5.07 (s, 1H, OH exchangeable by D_2_O), 5.78 (s, 2 H, NH_2_ exchangeable by D_2_O), 7.78–9.07 (m, 8 H, CH-Ar), 13.11 (s, 1H, NH exchangeable by D_2_O). ^13^C NMR (101 MHz, DMSO-*d*_*6*_) 12.2 (CH_3_), 55.5 (CH_2_), 59.3 (CH_2_), 124.7-146.9 (C-aromatic), 128.8 (*C* = N-NH), 149.8 (C = N of pyrazolone), 153.0 (C = O). Anal.Calc for C_21_H_18_N_6_O_11_S_2_ (481.76): Calcd: C: 44.90; H: 3.98; N: 14.55; S: 13.32. Found: C: 44.83; H: 3.92; N: 14.43; S: 13.43, MS (*m/z*): 481.71 [M^·+^].

##### Synthesis of 2-((4-(2-(1-(4-((4,6-dichloro-1,3,5-triazin-2-yl)amino)phenyl)-3-methyl-5-oxo-1,5-dihydro-4 H-pyrazol-4-ylidene)hydrazineyl)phenyl) sulfonyl)ethyl hydrogen sulfate (**4**)

For one hour, acetone (25 mL) and cyanuric chloride (1.85 g, 0.01 mol) were combined at a temperature below 5 °C. After that, over an hour, a neutral compound **3b** solution (4.81 g, 0.01 mol) in a 10% w/v aqueous sodium carbonate solution was slowly added to the stirred mixture. Additionally, a 1% w/v sodium carbonate solution was added to maintain the pH at neutral. For four hours, the reaction mixture was constantly stirred between 0 °C and 5 °C until a clear product was obtained. The resultant solution was filtered, washed with cold water, and finally dried.

Brown powder, yield: 91% (5.74 g), m.p: over 360 °C. IR (KBr, cm^− 1^) 3450 (OH), 3187 (NH), 3052 (Ar-H), 2931 (Aliph-H), 1640 (C = O), 1588 (C = N), 1478 (C = C), 1129 (S = O), 802 (C-Cl). ^1^H NMR (500 MHz, DMSO-*d*_*6*_) *δ* 2.23 (s, 3 H, CH_3_), 3.37 (t, 2 H, CH_2_), 3.74 (t, 2 H, CH_2_), 4.99 (s, 1H, OH exchangeable by D_2_O), 6.14 (s, H, NH exchangeable by D_2_O), 7.58–8.21 (m, 8 H, CH-Ar), 12.97 (s, 1H, NH exchangeable by D_2_O). ^13^C NMR (101 MHz, DMSO-*d*_*6*_) 12.3 (CH_3_), 55.5 (CH_2_), 58.4 (CH_2_), 117.3-150.4 (C-aromatic), 128.9 (*C* = N-NH). 151.3 (C = N of pyrazolone), 156.7 (C = O), 166.9 (C-Cl of triazine ring), 169.3 (N = *C*-N of triazine ring). Anal.Calc for C_21_H_18_Cl_2_N_8_O_7_S_2_ (627.44): Calcd: C: 40.07; H: 2.88; Cl: 11.26; N: 17.80; S: 10.19. Found: C: 40.13; H: 2.82; Cl: 11.23; N: 17.87; S: 10.23, MS (*m/z*): 627.32 [M^·+2^].

### Synthesis of nano-scale disperse reactive dyes **3a**, **3b**, and **4**

Nanoscale dyes **3a**, **3b**, and **4** were produced by ball milling (VQ-N High Energy Ball Mill, worldwide USA). For 40 min at 1,200 rpm, eight grinding balls were used to charge and dry mix dyes **3a**, **3b**, and **4** into 80 mL of stainless-steel agar. Each nano dye was assessed using a JEM-2100 High Resolution Transmission Electron Microscope (JEOL-100 SX, Japan).

### Treating polyester with chitosan following sodium hydroxide treatment

Polyester fabric was alkalized for 40 min at 90 °C in an Ahiba apparatus using an alkali concentration of 15 g/l and an aqueous sodium hydroxide solution. 75% purity chitosan from Sigma-Aldrich with low molecular weight was used without additional purification in a freshly prepared solution at a concentration of 1 g/l. At room temperature, the liquor ratio in the chitosan treatment was 1:20 after 20 min of continuous stirring. After treatment, the samples were washed with distilled water, dried in ambient air, and then dried again^3,27,28^.

### Measurement of the dyeing properties

#### Traditional procedure for dyeing different fabrics with new nano dyes (**3a**, **3b**, and **4**)

**Fig. 1:**
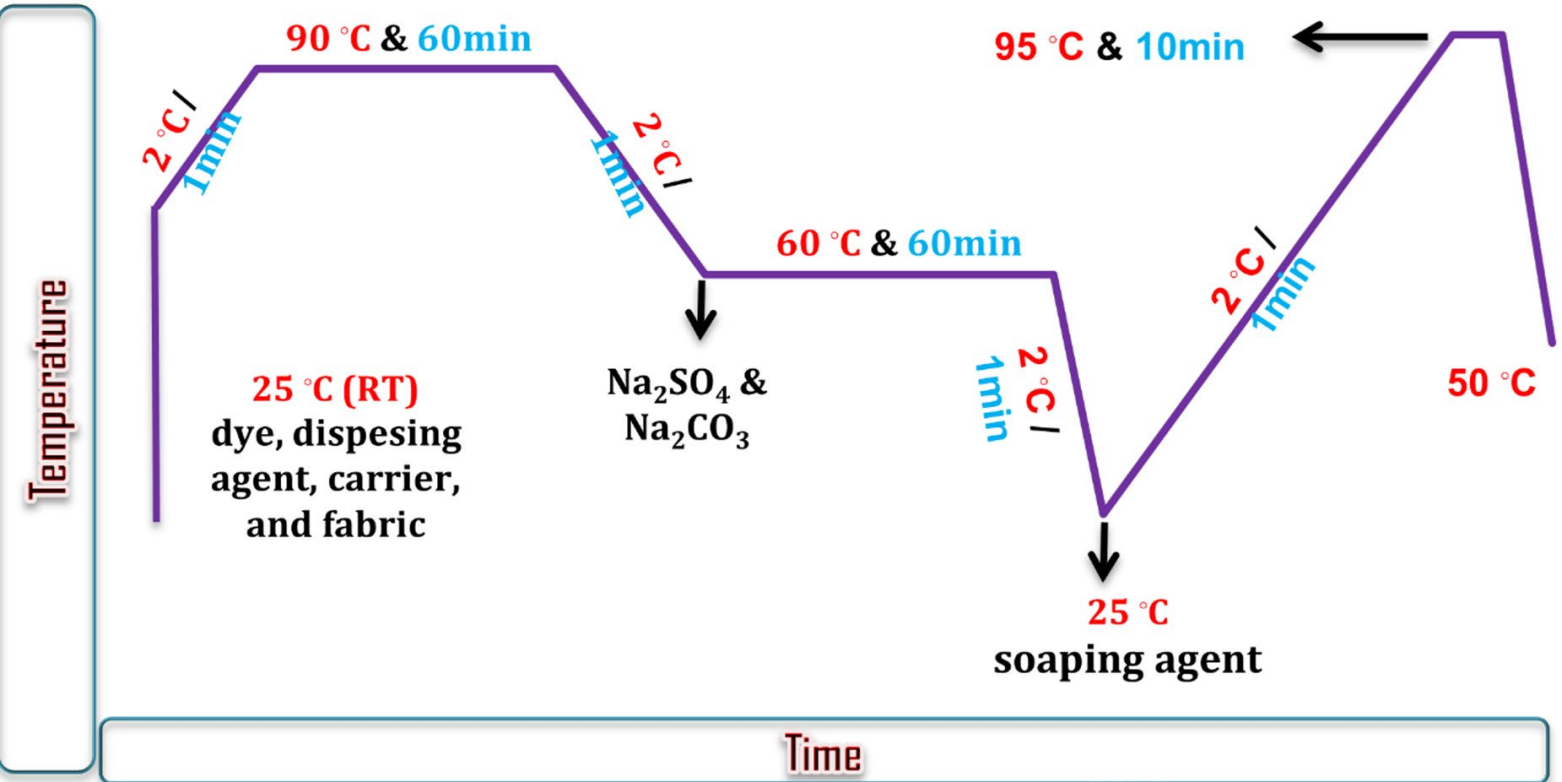
One bath dyeing profile of all fabrics with partially solubilized disperse reactive dyes.

The National Research Center in Cairo, Egypt, conducted all testing and evaluations of the fastness properties of the synthetic dyes. The synthesized dyes were used to color polyester, cotton, wool, polyester/cotton blend (50:50), polyester/wool blend (55:45), and polyester/chitosan as a blend fabric. The dyeing procedures were followed in compliance with the established protocols^3,29^. To provide sufficient dispersion in the dye solution, the dyes were powdered using a carefully chosen dispersing agent (Sera Gal P-CHD, Daystar). Each fabric was dyed using distilled water in a 1:10 liquor ratio. The dye solution was made with 2% OWF (of fabric weight) and 1.5 g/l of carrier (Optinol MBF, Yorkshire) (Fig. 1).

#### Fastness properties

Several standardized techniques were used to evaluate the fastness characteristics of the dyed fabrics. The ISO105-C03 was followed for conducting the washing fastness test. An automatic Crockmeter was used to measure rubbing fastness using the ISO105-X12 technique. The Q-Sun Xenon Test Chamber was used to test lightfastness using the AATCC 16-2004 protocol. The test for perspiration fastness was ISO105-EO4 (1989). The sublimation fastness of the colored polyester samples was evaluated using the ISO 105-F04 method on a sublimation tester.

#### The K/S formula

The color strength (**K/S** value) is calculated from the reflectance (**R**) of the dyed fabric at the maximum absorption wavelength (**λ**_**max**_) as illustrated in Eq. (1). The percentage of dye fixation is generally calculated by measuring the color strength of the fabric before and after washing off unfixed dye, as in Eq. (2)^30^.

1 $$K/S=(1-R)^{2}/2R$$

K: The adsorption coefficient of the dyed samples. S: The scattering coefficient.

R: The decimal fraction of reflectance of the dyed samples.

#### Fixation rate calculation (F%)

2 $${\text{Fixation Rate}} (F\%)={{(K/S)_{washed}/(K/S)_{unwashed}}} \times 100$$

(K/S)_washed_ and (K/S)_unwashed_ are the K/S values of the dyed fabric after washing by soap to remove unfixed dyes and after drying, before soaping, respectively.

### Antimicrobial profiling of nanostructured pyrazolone-based disperse reactive dyes

#### Microbial test panel

This study evaluated the bioactive potential of the synthesized dyes against a panel of six clinically relevant bacterial isolates. The Gram-negative cohort consisted of *Escherichia coli* (ATCC 25922), *Klebsiella pneumoniae* (ATCC 13883), and *Pseudomonas aeruginosa* (ATCC 27853). Gram-positive representatives included *Staphylococcus epidermidis* (ATCC 12228), *Staphylococcus haemolyticus* (ATCC 29970), and methicillin-resistant *Staphylococcus aureus* (MRSA, ATCC 43300). All bacterial strains were procured from the archival culture repository of the Bacteriology Unit, Department of Botany and Microbiology, Faculty of Science, Tanta University, Egypt. Additionally, the pathogenic yeast *Candida albicans* (ATCC 10231) was incorporated into the assay, obtained from the cognate Mycology Unit within the same department.

#### Determination of antimicrobial susceptibility profiles

The antibiotic resistance profiles of the clinical bacterial isolates were delineated *via* the standardized Kirby-Bauer disc diffusion assay^31,32^. In brief, standardized suspensions (0.5 McFarland standard) of each strain were uniformly inoculated onto Mueller-Hinton agar plates. Subsequently, a panel of eleven antibiotic-impregnated discs (Oxoid, England), representing distinct mechanistic classes (Table 1), was aseptically applied to the seeded agar surface. Following a 24 h incubation at 37 °C, the diameters of the resulting growth inhibition zones were accurately measured. These values were interpreted as sensitive (S), intermediate (I), or resistant (R) according to established Clinical and Laboratory Standards Institute breakpoints^33^, with data compiled in Table 2.

**Table 1:** Antibiotic discs and their disc concentrations.

Antibiotic class	Antimicrobial agent	Code	Conc. (µg/disc)
Aminoglycosides	Amikacin	Ak	10
β-Lactamase inhibitors	Amoxicillin/clavulanic acid	AMC	20/10
Carbapenems	Imipenem	IPM	10
	Meropenem	MEM	10
Cephalosporins	Cephalexin	CE	30
	Ceftriaxome	CRO	30
	Cefotaxime	CTX	30
Macrolides	Erythromycin	E	15
Penicillins	Ofloxacin	OFX	5
Quinolones	Norfloxacin	NOR	10
	Ciprofloxacin	CIP	10

**Table 2:** In vitro screening of the selected bacterial strains’ resistance to 11 tested antibiotics.

Bacterial strain	Antibiotic resistance pattern	Antibiotic resistance	
		No.	%
S. epidermidis	AMC and CE	2	18.18
S. haemolyticus	AK, NOR, IPM, CE, E, CRO, MEM, OFX, CTX, and CIP	10	90.91
E. coli (ATCC)	AK, AMC, CE, E, and CTX	5	45.45
MRSA	AK, AMC, CE, CRO, and CTX	5	45.45
K. pneumoniae	AK, NOR, IPM, AMC, CE, E, CRO, MEM, OFX, CTX, and CIP	11	100.00
P. aeruginosa	AK, NOR, AMC, CE, E, CRO, CTX, and CIP	8	72.73

#### Antifungal susceptibility profiling of *Candida albicans*

The susceptibility of the *C. albicans* isolate to a panel of antifungal agents was assessed using a modified Kirby-Bauer disk diffusion assay on Sabouraud Dextrose Agar (SDA), as per the methodology of Jabeen et al.^34^. The panel comprised commercially sourced disks (Himedia, India) impregnated with polyenes (nystatin, amphotericin B) and azoles (fluconazole, itraconazole, ketoconazole, voriconazole). A standardized inoculum (0.5 McFarland standard) was uniformly seeded onto the agar surface. Following aseptic placement of the antifungal disks, plates were incubated at 28 °C for 24–48 h. Resultant zones of inhibition were measured and categorized as susceptible, intermediate, or resistant based on established interpretive criteria^35,36^, with data compiled in Table 3.

**Table 3:** In vitro screening of *C. albicans* resistance to the investigated antifungals.

Antifungal resistant pattern	Antifungal resistance	
Amphotericin B, fluconazole, nystatin, and itraconazole	No.	%
	4	66.67

#### Evaluation of antimicrobial activity

The bioactive potential of the synthesized nanostructured dyes against the designated microbial panel was quantified using a standardized agar well diffusion assay^36^. Bacterial and fungal (*C. albicans*) lawns were prepared by uniformly inoculating nutrient agar and Sabouraud dextrose agar plates, respectively, with standardized suspensions (approximately 6 × 10^6^ CFU/mL). Subsequently, equidistant wells were aseptically excised from the solidified agar using a sterile cork borer. Each well was loaded with 50 µL of the target compound, formulated as a 60 mg/mL solution in dimethyl sulfoxide (DMSO). Following compound application, bacterial plates were incubated at 37 °C for 24 h, while fungal plates were incubated at 28 °C for the same duration. Antimicrobial efficacy was quantified by measuring the diameters (mm) of the resulting clear zones of growth inhibition.

#### Quantitative assessment of antimicrobial efficacy by colony-forming unit (CFU) assay

To provide quantitative standard data on the antimicrobial efficacy of the most potent dye 3b, a CFU assay was performed according to established protocols^37^. Three representative pathogens were selected: Gram-positive (*S. haemolyticus* ATCC 29970), Gram-negative (*K. pneumoniae* ATCC 13883), and fungal (*C. albicans* ATCC 10231). Overnight cultures were adjusted to approximately 1 × 10^6^ CFU/mL. Each microbial suspension (1 mL) was treated with compound 3b (60 mg/mL in DMSO, final concentration 100 µg/mL) and incubated at 37 °C (bacteria) or 28 °C (*C. albicans*) for 4 h. DMSO (1%) served as the negative control. After incubation, serial dilutions were prepared and plated onto nutrient agar (bacteria) or Sabouraud dextrose agar (*C. albicans*). Plates were incubated for 24–48 h, and viable colonies were counted. The initial load (at zero time) was determined by plating the untreated suspension without incubation. All experiments were performed in triplicate, and results are expressed as mean log_10_ CFU/mL ± SD.

## Results and discussion

### Chemistry

**Scheme 1:**
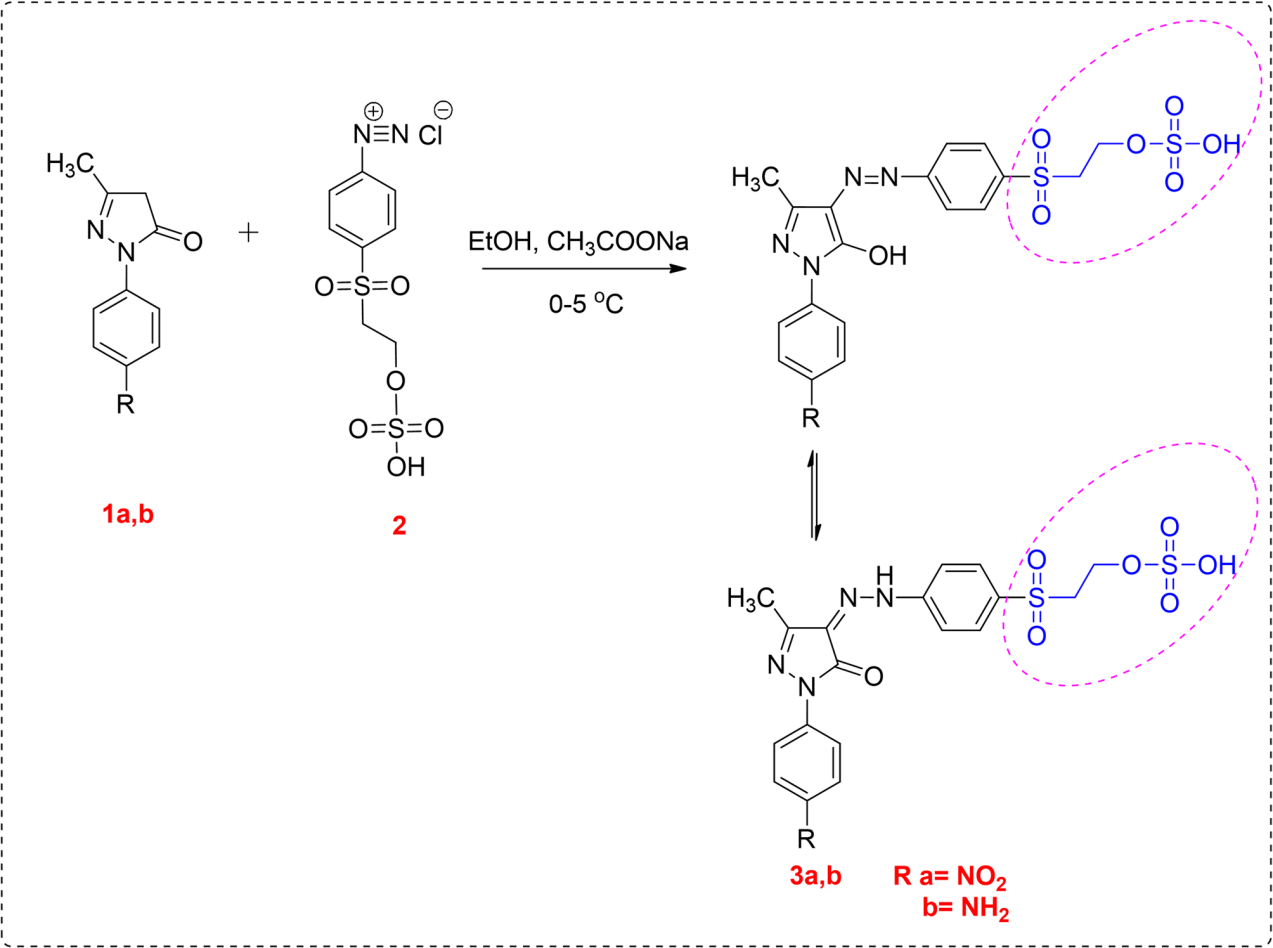
Synthesis of disperse reactive dyes (**3a**,** 3b**) with one reactive center.

**Scheme 2:**
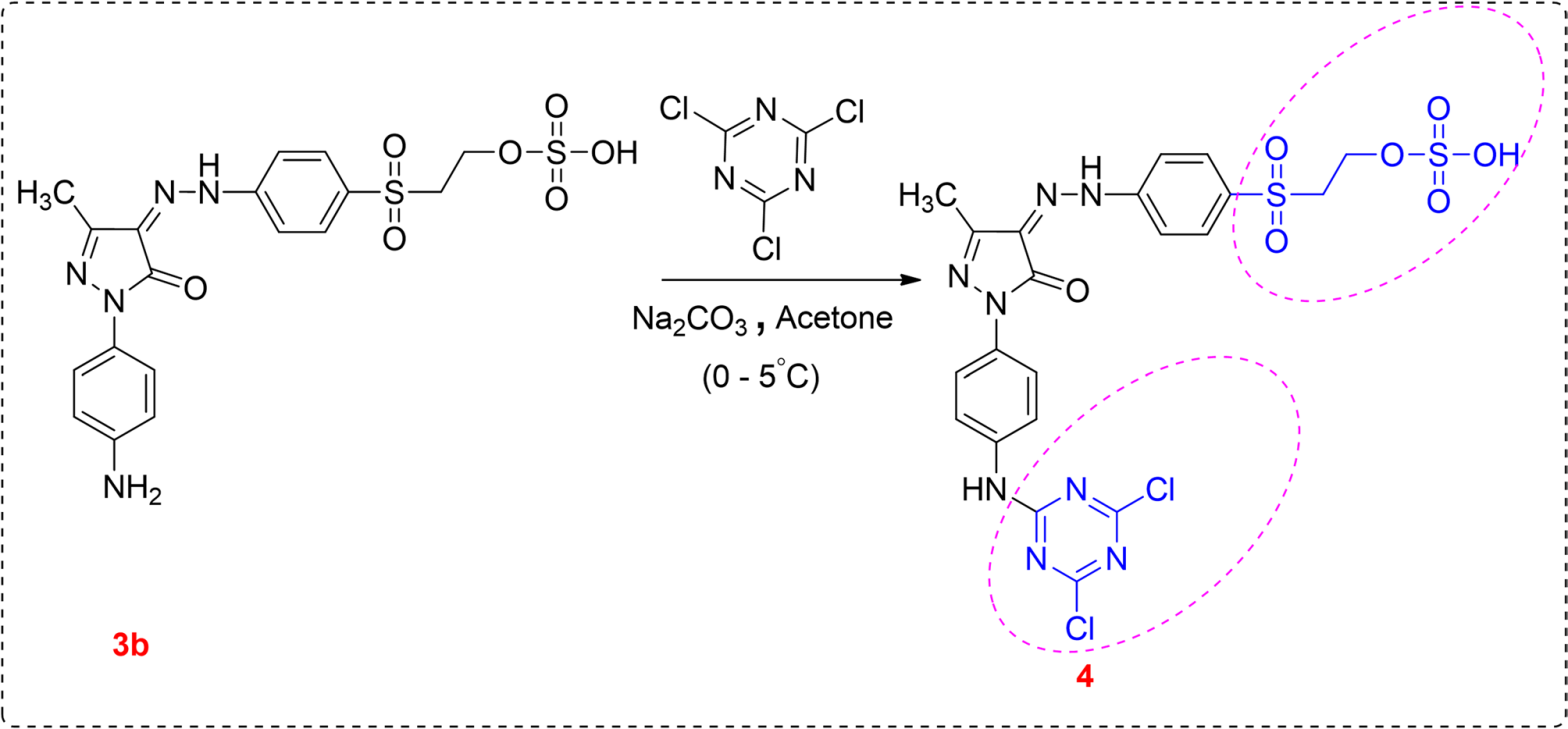
Synthesis of disperse reactive dye (**4**) with two reactive centers.

This study is a part of our program toward the synthesis of novel vinyl sulfone dispersible reactive dyes based on the pyrazolone moiety containing one or two reactive centers. The compounds **3a** and **3b**, which have one reactive center, were synthesized by treatment of the diazonium salt compound **2** with (4-nitro/amino)-1-phenyl)-3-methyl-1*H*-pyrazol-5(4*H*)-one (**1a/1b**) in ethanol and ethyl acetate as a green method (Scheme 1). On the other hand, the reaction of compound **3b** with cyanuric chloride in acetone and sodium carbonate gave 2-((4-(2-(1-(4-((4,6-dichloro-1,3,5-triazin-2-yl)amino)phenyl)-3-methyl-5-oxo-1,5-dihydro-4*H*-pyrazol-4-ylidene)hydrazineyl)phenyl)sulfonyl)ethyl hydrogen sulfate (**4**), which has two reactive centers (Scheme 2). All synthesized dyes **3a**, **3b**, and **4** were converted to nano form by using the high-energy ball milling technique. Using different spectrum studies, the structures of the prepared compounds were confirmed. From the IR spectra of compounds **3a**, **3b**, and **4**, new bands appeared at *ν* 3450–3481, 3117–3230, and 1562–1640 cm⁻¹ for OH, NH, and C = O groups, respectively. Further, the appearance of the bands of NH elucidates the presence of synthesized dyes in hydrazo form. Moreover, the ^1^H NMR spectra of dyes **3a**, **3b**, and **4** showed new triplet signals at *δ* 3.37–3.65 ppm and *δ* 3.37–3.82 ppm attributed to *CH₂*SO₂ and *CH₂*OSO_3_H, respectively, and a new singlet signal appeared at *δ* 4.99–5.42 ppm for OH, which is exchangeable by D₂O. Also, the appearance of a new singlet signal at *δ* 12.97–13.20 ppm for NH and exchangeable by D₂O, which elucidates the hydrazo form of the synthesized dyes. In addition, the ^13^C NMR spectra gave signals at *δ* 12.2–12.8, 55.0-55.5, 57.8–59.3, 127.6-128.9, and 153.0-156.7 ppm attributed to CH_3_, *CH₂*SO₂, *CH₂*OSO_3_H, *C* = NNH, and *C* = O, respectively. The molecular ion peaks in the mass spectra matched the appropriate molecular formulas of the produced substances. The computed results showed a correlation with the elemental analysis’s observed values.

**Fig. 2:**
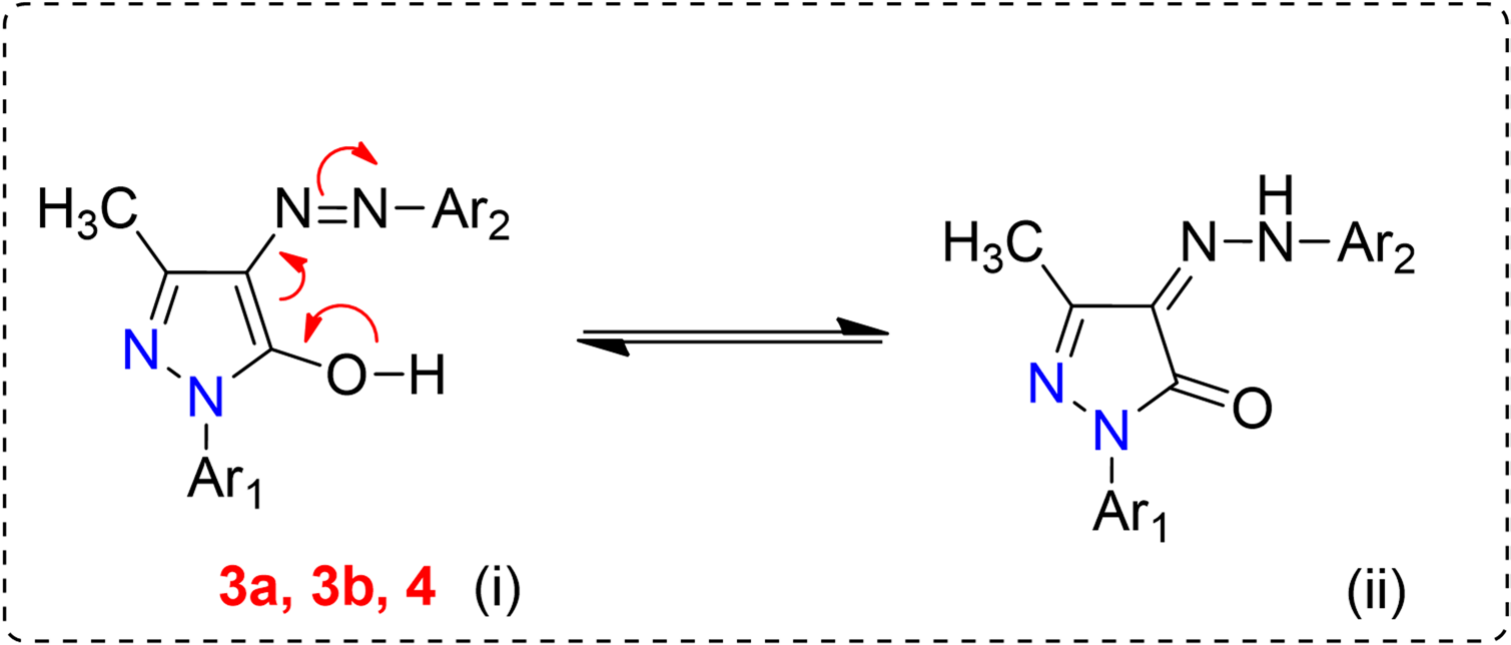
Mechanism of the hydrazo form of compounds **3a**,** 3b**, and **4**.

From the above data of ^1^H NMR and ^13^C NMR spectra, we ensure that the structure of all the synthesized dyes is in hydrazo form (Fig. 2).

### Characterization of the newly synthesized nano dyes 3a, 3b, and 4

The high-energy ball milling technique utilized for the preparation of nano-disperse dyes was a suitable method and produced the desired crystalline size. According to XRD data (Supplementary part), the crystalline size of nano dyes was from 7.39 to 22 nm. The average crystallite size (D) was calculated using Scherer’s equation: D = 0.9λ/(h½ cosθB), where θ refers to the diffraction angle, h½ refers to the diffraction peak’s whole width at half maximum (in radians), and λ is the wavelength for Cu-Kα (λ = 1.5405). Because XRD gives us only information about crystalline size and does not provide information on the size distribution, TEM was used to determine the shape and size distribution of the nanoparticles. (Table 4) provides the XRD crystallite size as well as the TEM particle size. The low value particle size of the prepared nano-disperse dyes supports the benefit of using the ball milling technique for this purpose.

**Table 4:** Crystalline and particle size of nano-disperse dyes **3a**,** 3b**, and **4**.

Nano-disperse reactive dye	Crystalline size nm (from XRD)	Particle size nm (from TEM)
**3a**	± 7.39	± 6.86
**3b**	± 8.37	± 7.88
**4**	± 22	± 19.85

**Fig. 3:**
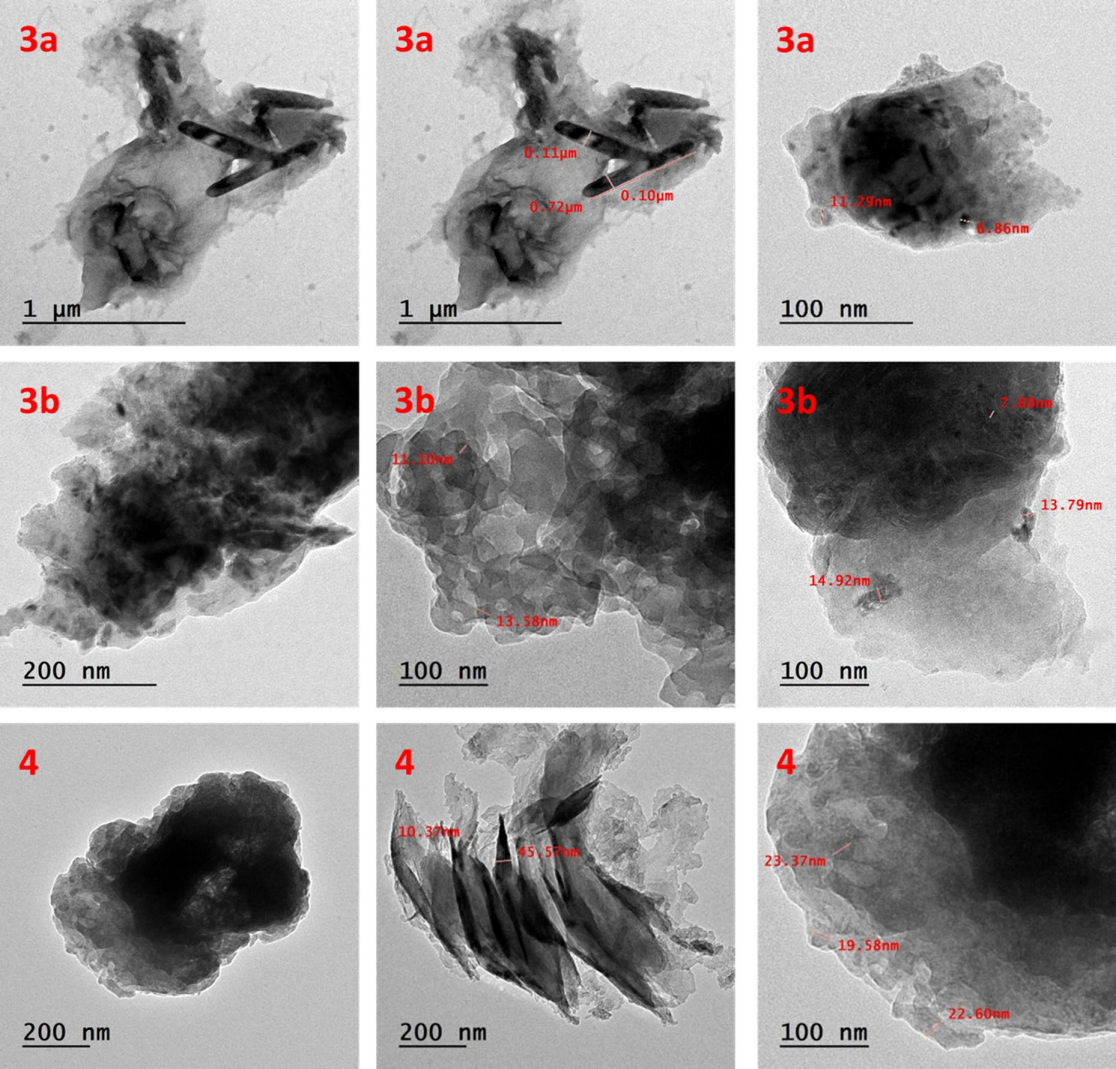
TEM of **3a**, **3b**, and **4**.

**Fig. 4:**
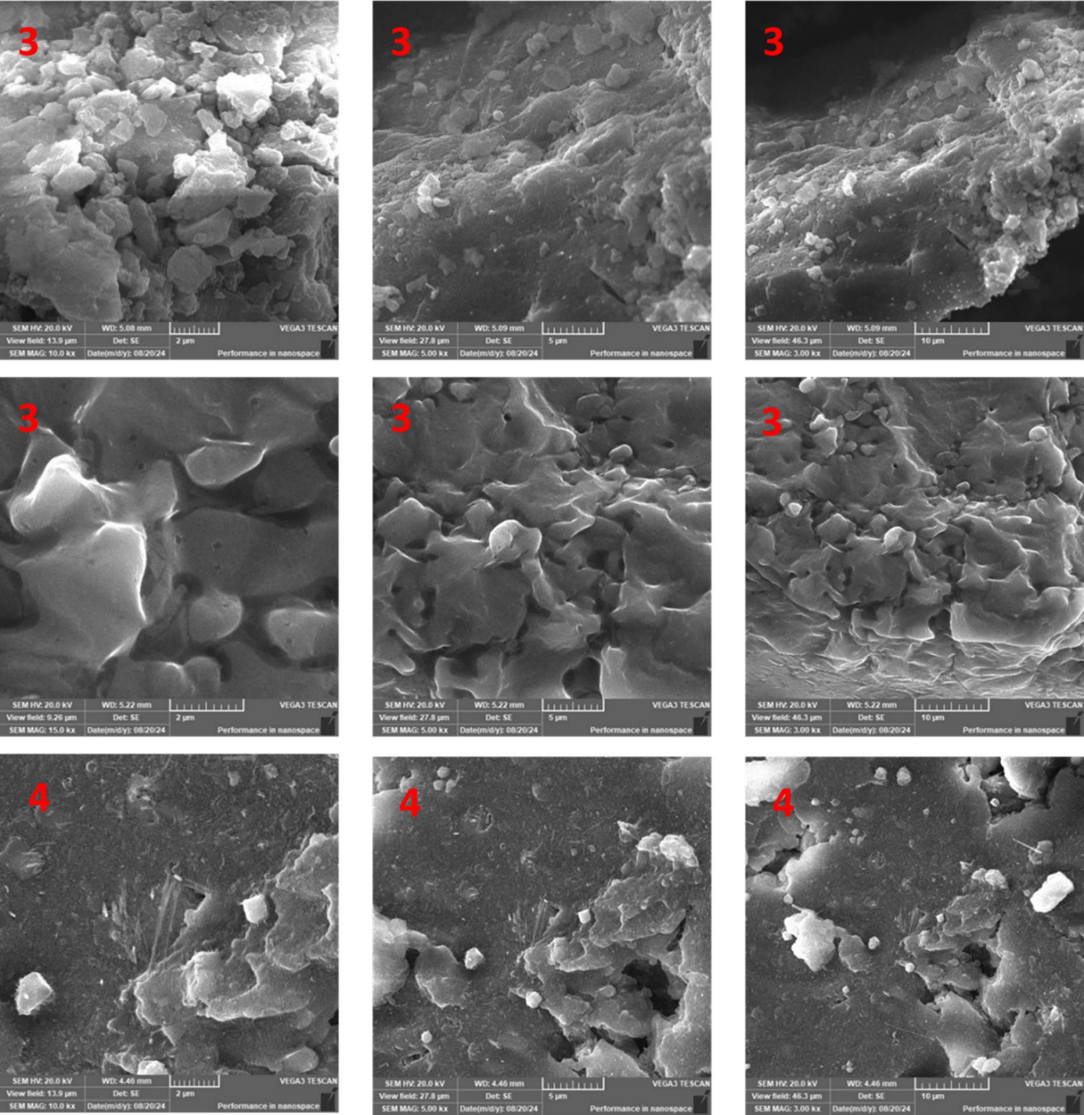
SEM of **3a**, **3b**, and **4**.

Figure 3 shows transmission electron microscope (TEM) investigation of nano-disperse dyes **3a**, **3b**, and **4**. TEM showed a nanoparticle or agglomerate of nanoparticles. For **3a**, the investigations showed a nanoparticle with 6.86 nm, which is in accordance with the particle size calculated from XRD (7.39 nm). Also, for **3b**, the particle size calculated from TEM was 7.88 nm, whereas it was 8.37 nm from XRD calculations. In addition, the particle size of **4** was found to be 19.58 nm, as investigated from TEM images, and was 22 nm from XRD calculations. (Fig. 4) shows scanning electron microscope (SEM) of **3a**,** 3b**, and **4**. SEM investigation was carried out at different magnifications, including high and low magnifications. These images show a homogeneous, smooth surface and provide crucial information about the surface morphology of the produced nano dyes.

### Evaluation of fastness and K/S% parameters of different fabrics at traditional conditions

The color fastness of disperse reactive dyes **3a**,** 3b**, and **4** on various fabrics was tested at a temperature of 90 °C; pH was then adjusted to 4.5-5 using diluted acetic acid, and the temperature was maintained for 60 min before being cooled to 60 °C to improve the fixation of the disperse reactive dyes on cotton, polyester/cotton blend (50:50), and polyester/chitosan fabrics. 50 g/l of sodium sulfate (Na_2_SO_4_) and 25 g/l of an alkali solution (Na_2_SO_4_) were added after an extra 30 min at 60 °C; the sulfonyl ethyl hydrogen sulfate group had been converted into a vinyl sulfone group in this step, which then created a covalent bond with the hydroxyl group of both pure cotton and the polyester/cotton and chitosan blend; on the other hand, wool and a polyester/wool blend (55:45) were kept at 60 °C for an extra half hour, except for the polyester fiber; after being rinsed, the dyed fiber were soaked for ten minutes at 95 °C using 1.5 g/l of a soaping agent, and then they were allowed to dry at room temperature. It also depends on whether the dyes have an (EWG) (NO_2_) or (EDG) (NH_2_) and/or if they have one or two reactive groups (vinyl sulfone/cyanuric chloride) (Table 5). Also, the sublimation fastness technique, which shows the dyes’ resistance to high temperatures and pressure, was used to determine color fastness to sublimation. As seen in Table 6, the dyes **3a**, **3b**, and **4** demonstrated very good to remarkable performances (**4**). We noticed that all fastness properties (light, washing, rubbing, and perspiration fastness) gave excellent results except light and wet rubbing, which gave moderate to good results. In contrast, when dyes **3a** and **3b** with one reactive center were compared, we found that dye **3a** has high K/S% values in all types of fabric, for example, in PET/W, the K/S% for **3a** and **3b** is 23.27 and 11.39, respectively. We discovered that dye **3a** with an EWG (NO₂) has better substantivity than dye **3b** with an EDG (NH₂). This is because the NO₂ group reduces electron density on the carbon atom at the reactive core. The decrease in electron density makes the carbon atom at the reactive center more vulnerable to attack by the nucleophilic fabric anion (Cell-OH). Thus, it achieves a high degree of fixation for dye **3a**. While comparing dye **3b**, which has one reactive center (vinyl sulfone), and dye **4**, which has two reactive centers (vinyl sulfone and cyanuric chloride), we found that the value of K/S% is higher in the bi-functional dye **4** (K/S% = 16.37) than in the mono-functional dye **3b** (K/S% = 11.7) in wool fabric, as an example. This is because bi-functional dyes can form bonds in two locations. Which means that even if one reactive group hydrolyzes (reacts with water), there is still a good chance that the second group will form a strong covalent bond with the fabrics. On the other hand, the bi-functional reactive dyes don’t decrease the rate of hydrolysis of the dyes themselves; rather, it often increases the overall efficiency of fixation, making the dyes less sensitive to the wastage caused by hydrolysis compared to traditional mono-functional dyes, greatly increasing the dyeing efficiency and fastness characteristics (See supplementary part)^38,39^.

**Table 5:** Color parameters of the synthesized nano dyes **3a**,** 3b**, and **4** onto the pure, blend, and modified fabrics at traditional conditions^a^.

Dye.no	Fabrics	K/S %	Light fastness	Rubbing fastness		Washing fastness				Perspiration fastness							
										Acidic				Alkaline			
				Wet	Dry	St	St_1_	St_2_	Alt.	St	St_1_	St_2_	Alt.	St	St_1_	St_2_	Alt.
**3a**	PET	4.76	6	3–4	4	4	4	4	4	4	4	4	4	4	4	4	4
	CO	7.49	6	3–4	3–4	4	4	4	4	4	4	4	4	4	4	4	4
	W	12.39	6	2–3	3	4	4	4	4	4	4	4	4	4	4	4	4
	PET/CS	20.77	6	3–4	3–4	4	4	4	4	4	4	4	4	4	4	4	4
	PET/CO	9.95	6	3	3	4	4	4	4	4	4	4	4	4	4	4	4
	PET/W	23.27	6	2–3	3	4	4	4	4	4	4	4	4	4	4	4	4
**3b**	PET	3.67	5	3–4	3–4	4	4	4	4	4	4	4	4	4	4	4	4
	CO	5.60	5	3–4	3–4	4	4	4	4	4	4	4	4	4	4	4	4
	W	11.70	6	2–3	3–4	4	4	4	4	4	4	4	4	4	4	4	4
	PET/CS	5.95	4–5	3–4	3–4	4	4	4	4	4	4	4	4	4	4	4	4
	PET/CO	5.59	4–5	3–4	4	4	4	4	4	4	4	4	4	4	4	4	4
	PET/W	11.39	4–5	3	4	4	4	4	4	4	4	4	4	4	4	4	4
**4**	PET	5.70	5	3–4	3–4	4	4	4	4	4	4	4	4	4	4	4	4
	CO	11.56	5	4	4	4	4	4	4	4	4	4	4	4	4	4	4
	W	16.37	6	3	3–4	4	4	4	4	4	4	4	4	4	4	4	4
	PET/CS	14.33	6	3–4	3–4	4	4	4	4	4	4	4	4	4	4	4	4
	PET/CO	11.24	6	4	4	4	4	4	4	4	4	4	4	4	4	4	4
	PET/W	14.87	6	3–4	4	4	4	4	4	4	4	4	4	4	4	4	4

**St**: Staining on cotton; **St**_**1**_: Staining on wool; **St**_**2**_: Staining on polyester; **Alt**: Alteration; **PET**: Polyester Fabric; **CO**: Cotton Fabric; **W**: Wool Fabric; **PET/CS**: Polyester modified Fabric; **PET/CO**: Polyester/Cotton blend Fabric (50%/50%); **PET/W**: Polyester/Wool blend Fabric (55%/45%). K/S% = (K/S)_washed_ /(K/S) _unwashed_ ×100: Color strength for dyed fabric after and before soaping, respectively.

^a^Rate for light fastness: 4–8 (acceptable), 1–3 (not acceptable); rate for different fastness: 3–5 (acceptable), 1–2 (not acceptable).

**Table 6:** The sublimation parameters for polyester fabric with dyes **3a**,** 3b**,** and 4**^a^

Dye.no	At 180 °C				At 210 °C			
	St	St_1_	St_2_	Alt.	St	St_1_	St_2_	Alt.
**3a**	4	4	4	4	4	4	4	4
**3b**	4	4	4	4	4	4	4	4
**4**	4	4	4	4	4	4	4	4

### Evaluation of K/S% parameters for different fabrics at different primary dyeing parameters

**Fig. 5:**
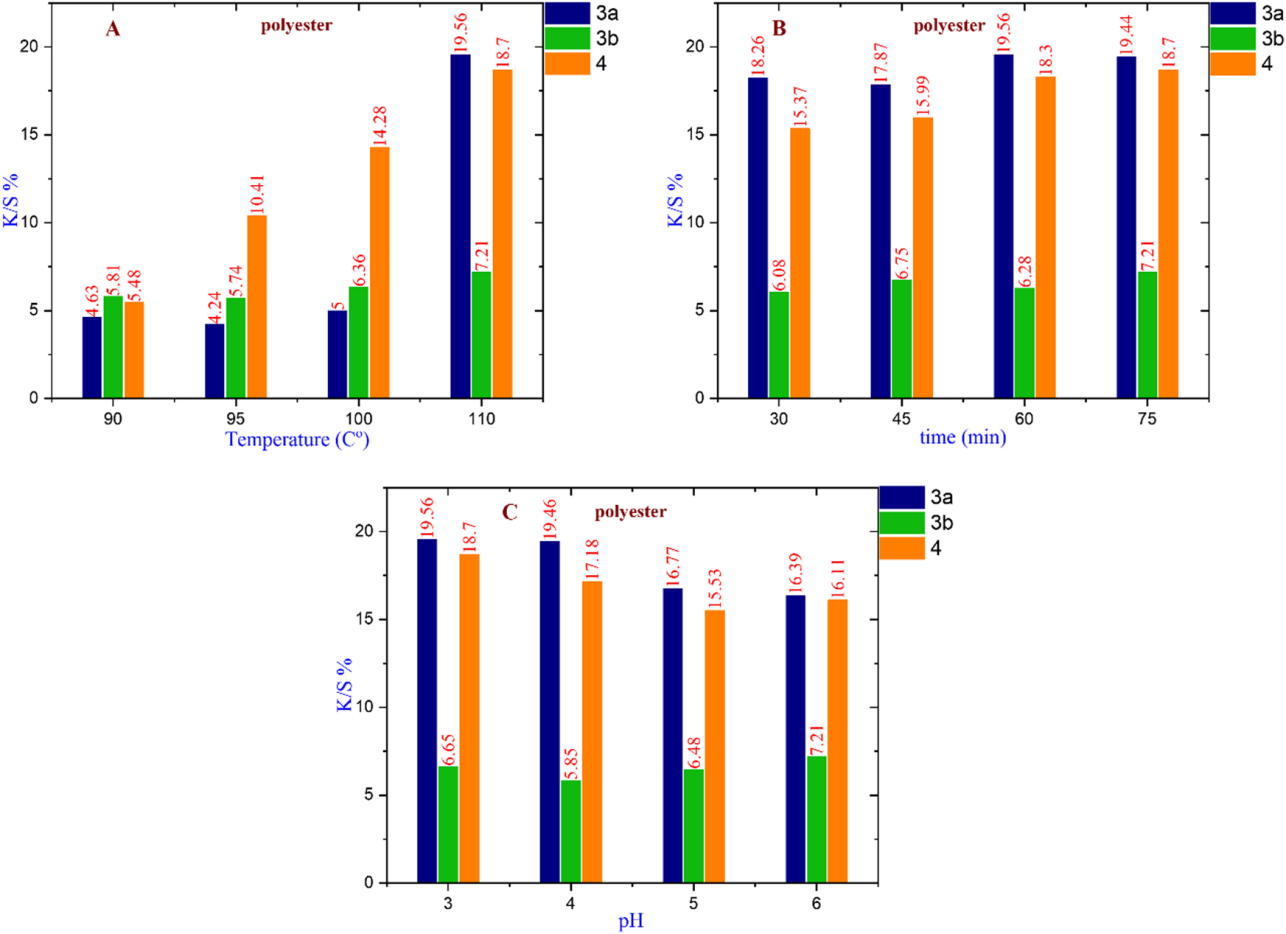
(**A**) The effect of changing temperature on compounds **3a** at (Time = 60 min & pH = 3), **3b** at (time = 75 min & pH = 6), and **4** at (time = 45 min & pH = 4). (**B**) The effect of changing time on compounds **3a** at (Temperature = 110 °C & pH = 3), **3b**, and **4** at (Temperature = 110 °C & pH = 6). (**C**) The effect of changing pH on compounds **3a** at (Temperature = 110 °C & time = 60 min), **3b** and **4** at (Temperature = 110 °C & time = 75 min).

**Fig. 6:**
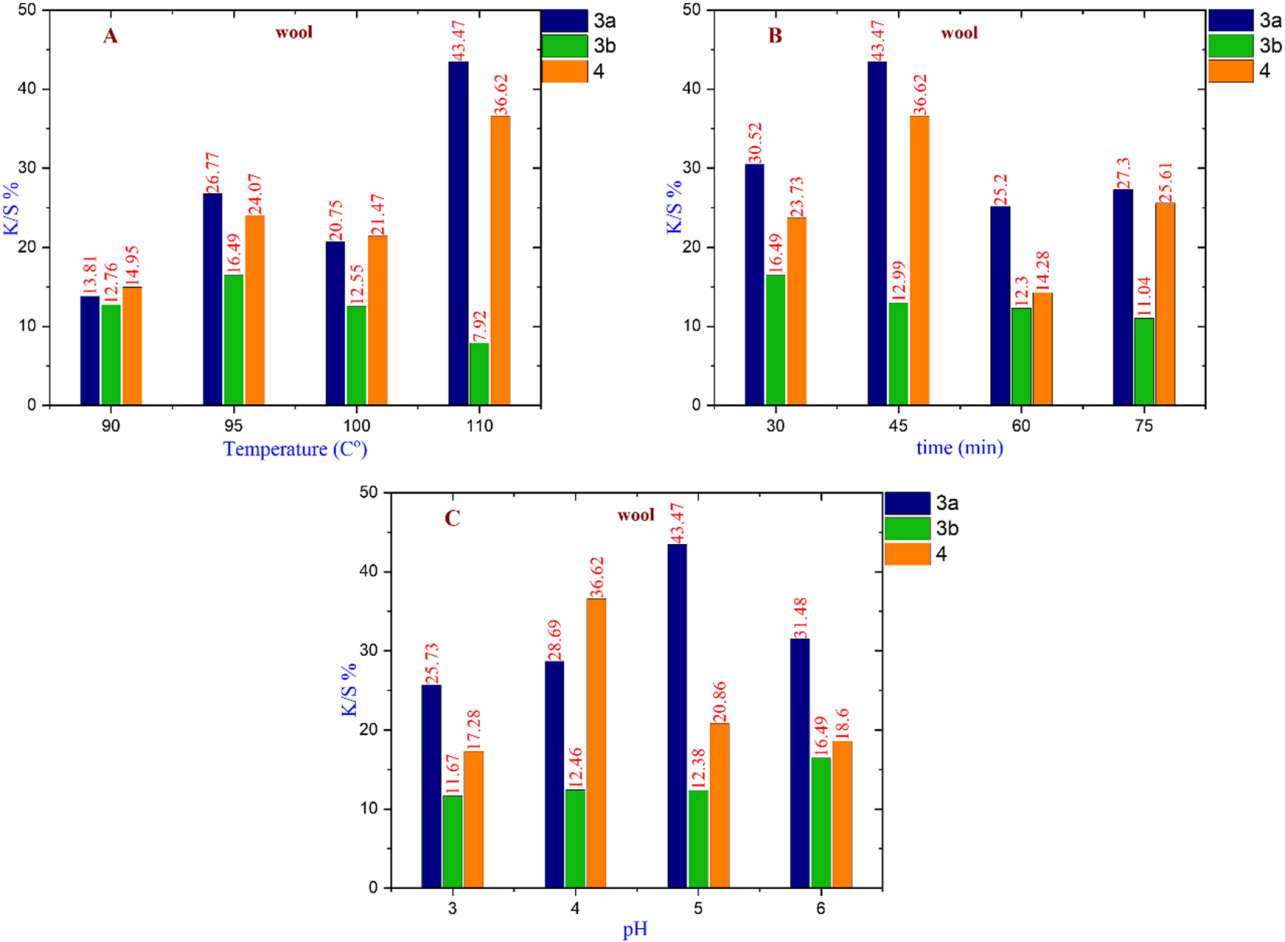
(**A**) The effect of changing temperature on compounds **3a** at (Time = 45 min & pH = 5), **3b** at (time = 30 min & pH = 6), and **4** at (time = 45 min & pH = 4). (**B**) The effect of changing time on compounds **3a** at (Temperature = 110 °C & pH = 5), **3b** at (Temperature = 95 °C & pH = 6), and **4** at (Temperature = 110 °C & pH = 4). (**C**) The effect of changing pH on compounds **3a**,** 4** (at Temperature = 110 °C & time = 45 min), and **3b** (at Temperature = 95 °C & time = 30 min).

**Fig. 7:**
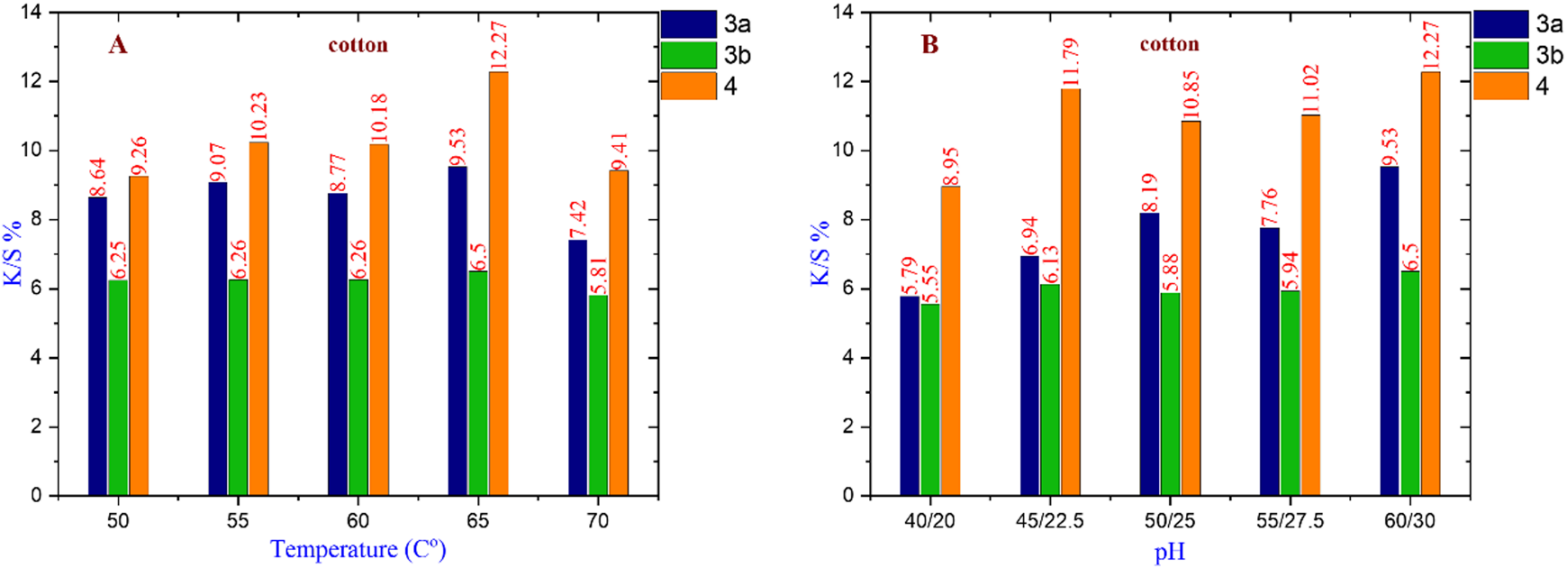
(**A**) The effect of changing temperature on compounds **3a**,** 3b**, and **4** (at Na_2_SO_4_ = 60 g/l & Na_2_CO_3_ = 30 g/l). (**B**) The effect of changing pH on compounds **3a**,** 3b**, and **4** (at Temperature = 65 °C).

**Fig. 8:**
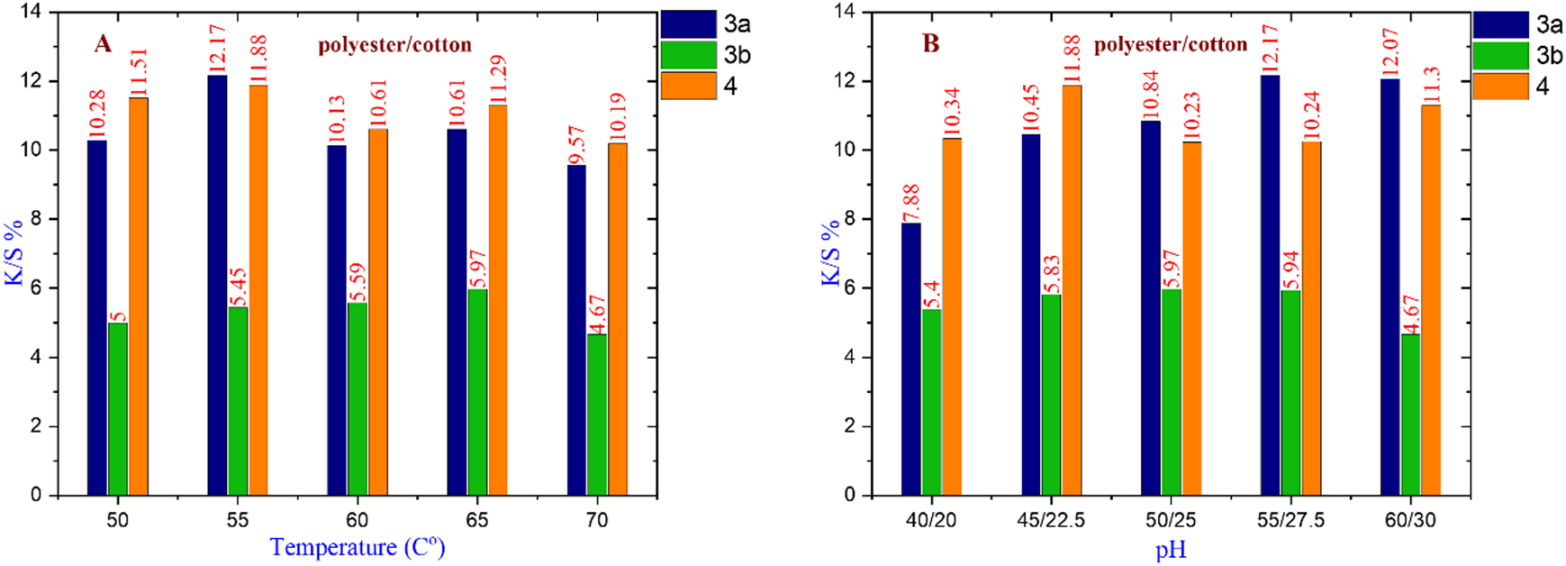
(**A**) The effect of changing temperature on compounds **3a** at (Na_2_SO_4_ = 55 g/l & Na_2_CO_3_ = 27.5 g/l), **3b** at (Na_2_SO_4_ = 50 g/l & Na_2_CO_3_ = 25 g/l), and **4** at (Na_2_SO_4_ = 45 g/l & Na_2_CO_3_ = 22.5 g/l). (**B**) The effect of changing pH on compounds **3a**,** 4** at (Temperature = 55 °C), and **3b** at (Temperature = 65 °C).

**Fig. 9:**
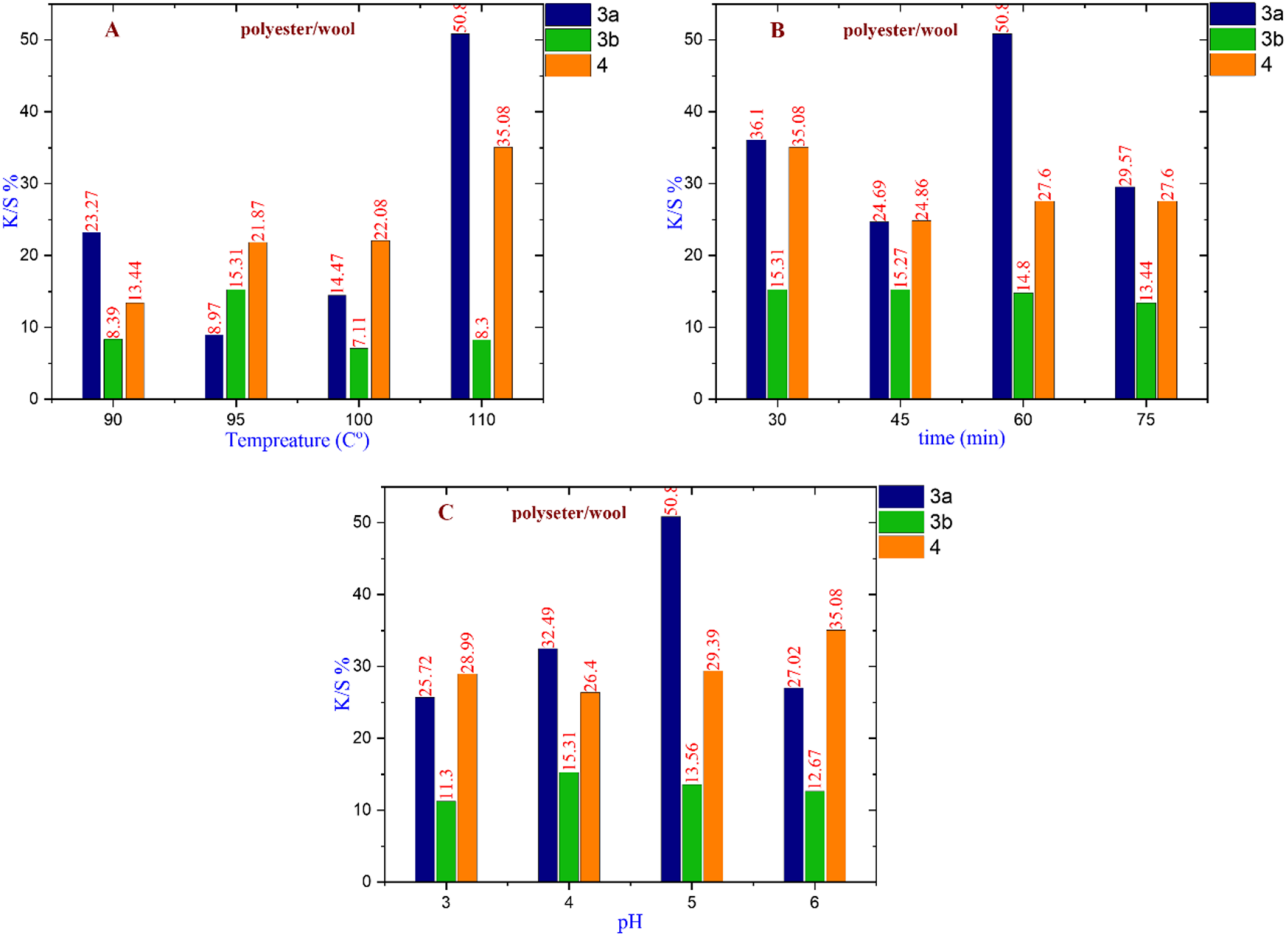
(**A**) The effect of changing temperature on compounds **3a** at (Time = 60 min & pH = 5), **3b** at (Time = 30 min & pH = 4), and **4** at (Time = 30 min & pH = 6). (**B**) The effect of changing time on compounds **3a** at (Temperature = 110 °C & pH = 5), **3b** at (Temperature = 95 °C & pH = 4), and **4** at (Temperature = 110 °C & pH = 6). (**C**) The effect of changing pH on compounds **3a** at (Temperature = 110 °C & time = 60 min), **3b** at (Temperature = 95 °C & time = 30 min), and **4** at (Temperature = 110 °C & time = 30 min).

**Fig. 10:**
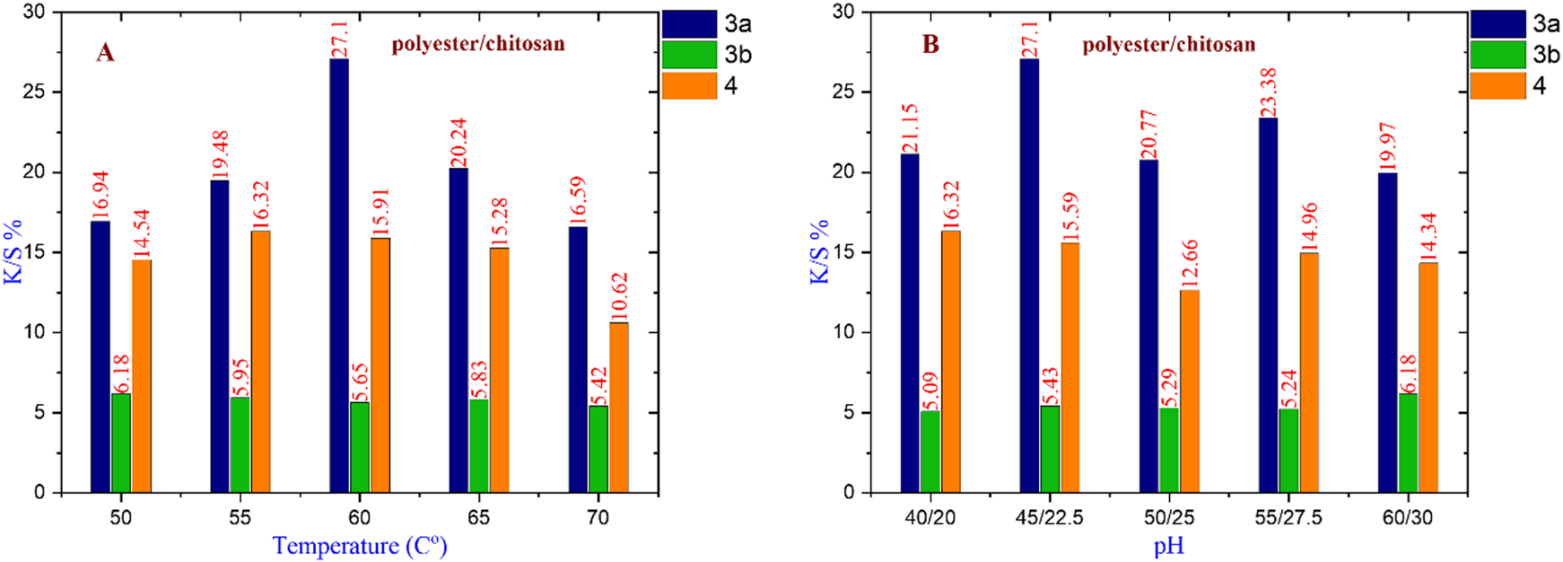
(**A**) The effect of changing temperature on compounds **3a** at (Na_2_SO_4_ = 45 g/l & Na_2_CO_3_ = 22.5 g/l), **3b** at (Na_2_SO_4_ = 60 g/l & Na_2_CO_3_ = 30 g/l), and **4** at (Na_2_SO_4_ = 40 g/l & Na_2_CO_3_ = 20 g/l). (**B**) The effect of changing pH on compounds **3a** at (temperature = 60 °C), **3b** at (temperature = 50 °C), and **4** at (temperature = 55 °C).

By changing the primary dyeing parameters of different fabrics, polyester, wool, and cotton as pure fabrics; polyester/wool and polyester/cotton as blended fabrics; and polyester/chitosan as a modified fabric, such as temperatures, pH levels, and time durations. We can identify the best K/S% value to achieve the optimal dyeing conditions. The nanoscale of dye particles and careful control of parameters like temperature, pH, and time duration for each type of fabric lead to higher dye exhaustion and fixation rates, which in turn reduce the amount of residual dye in the wastewater (Figs. 5, 6, 7, 8, 9 and 10).

#### For polyester fabric

Figure 5 demonstrates that dye **3a** exhibits the highest K/S% value of 19.56 at a temperature of 110 °C and a pH of 3 after 60 min. In contrast, dye **3b** achieves the highest K/S% value of 7.21 at 110 °C and a pH of 6 after 75 min. Additionally, dye **4** reaches a K/S% value of 18.70 at 110 °C and a pH of 3 after 75 min. Overall, dye **3a** is identified as the most appropriate nano-dispersed reactive dye for dyeing polyester fabric under the specified conditions.

#### For wool fabric

Figure 6 demonstrate that dye **3a** exhibits the highest K/S% value of 43.47 at a temperature of 110 °C and a pH of 5 after 45 min. In contrast, dye **3b** achieves the highest K/S% value of 16.49 at 95 °C and a pH of 6 after 30 min. Additionally, dye **4** reaches a K/S% value of 36.62 at 110 °C and a pH of 4 after 45 min. Overall, dye **3a** is identified as the most appropriate nano-dispersed reactive dye for dyeing wool fabric under the specified conditions.

#### For cotton fabric

Figure 7 show that dye **3a** has the maximum K/S% value of 9.53 at 65 °C, Na₂SO₄ 60 g/l, and Na₂CO_3_ 30 g/l. Dye **3b** has the greatest K/S% value of 6.50 at 65 °C with 60 g/l Na₂SO₄ and 30 g/l Na₂CO_3_. Dye **4** has a K/S% value of 12.27 at 65 °C with 60 g/l Na₂SO₄ and 30 g/l Na₂CO_3_. Overall, dye **4** is chosen as the best nano-dispersed reactive dye for coloring cotton cloth under the given conditions.

#### For polyester/cotton fabric

Figure 8 show that dye **3a** has the maximum K/S% value of 12.17 at 55 °C, Na₂SO₄ 55 g/l, and Na₂CO_3_ 27.5 g/l. Dye **3b** has the greatest K/S% value of 5.97 at 65 °C with 50 g/l Na₂SO₄ and 25 g/l Na₂CO_3_. Dye **4** has a K/S% value of 11.88 at 55 °C with 45 g/l Na₂SO₄ and 22.5 g/l Na₂CO_3_. Overall, dye **3a** is chosen as the best nano-dispersed reactive dye for coloring polyester/cotton cloth under the given conditions.

#### For polyester/wool fabric

Figure 9 demonstrates that dye **3a** exhibits the highest K/S% value of 50.85 at a temperature of 110 °C and a pH of 5 after 60 min. In contrast, dye **3b** achieves the highest K/S% value of 15.31 at 95 °C and a pH of 4 after 30 min. Additionally, dye **4** reaches a K/S% value of 35.08 at 110 °C and a pH of 6 after 30 min. Overall, dye **3a** is identified as the most appropriate nano-dispersed reactive dye for dyeing polyester/wool fabric under the specified conditions.

#### For polyester/chitosan fabric

Figure 10 show that dye **3a** has the maximum K/S% value of 27.10 at 60 °C, Na₂SO₄ 45 g/l, and Na₂CO_3_ 22.5 g/l. Dye **3b** has the greatest K/S% value of 6.18 at 50 °C with 60 g/l Na₂SO₄ and 30 g/l Na₂CO_3_. Dye **4** has a K/S% value of 16.32 at 55 °C with 40 g/l Na₂SO₄ and 20 g/l Na₂CO_3_. Overall, dye **3a** is chosen as the best nano-dispersed reactive dye for coloring polyester/chitosan cloth under the given conditions.

### FTIR spectra of undyed fabrics with new nano dyes 3a, 3b, and 4

**Fig. 11:**
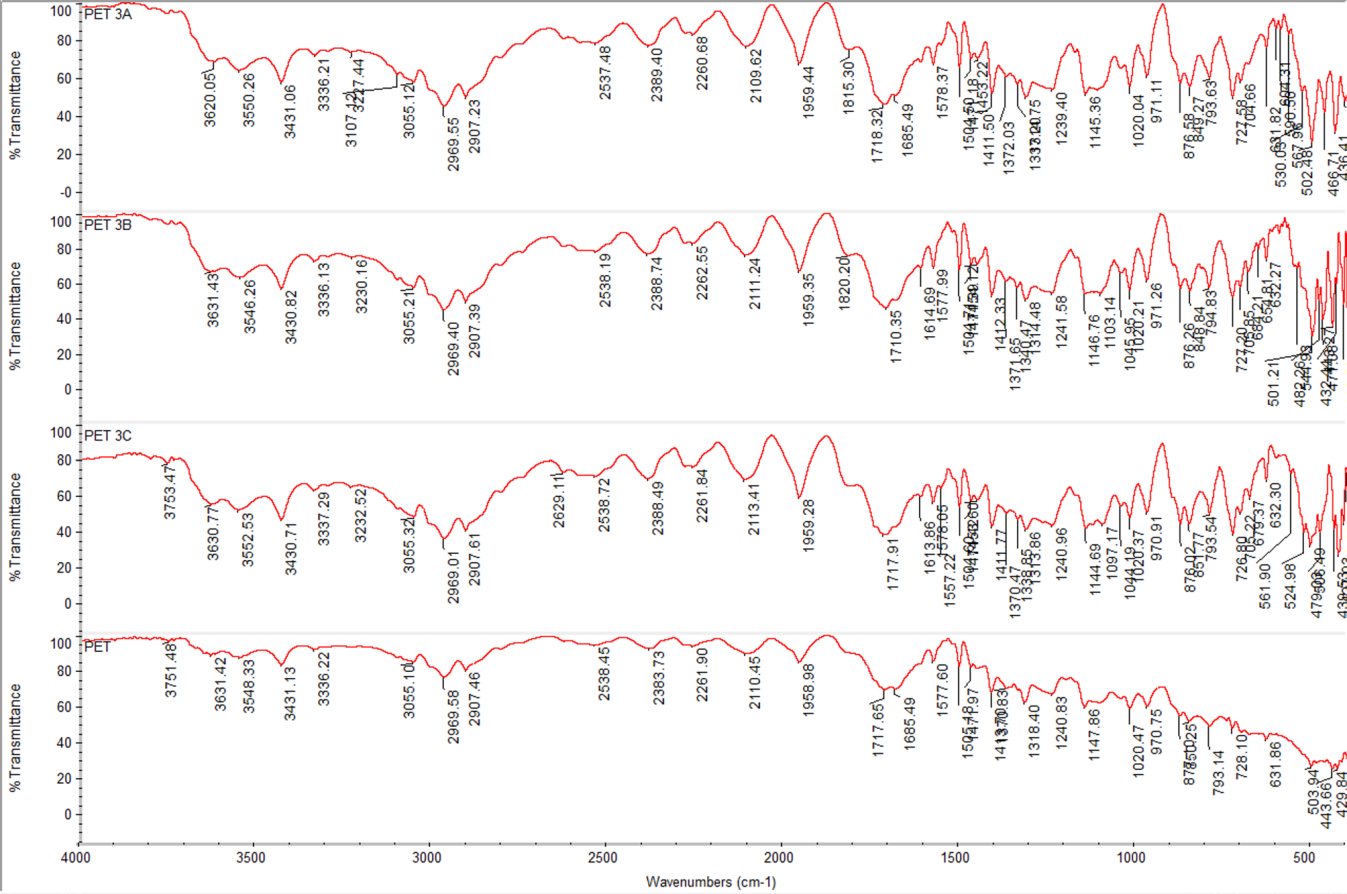
FTIR of undyed polyester fabric and dyed polyester fabric with dyes **3a**, **3b**, and **4**.

**Fig. 12:**
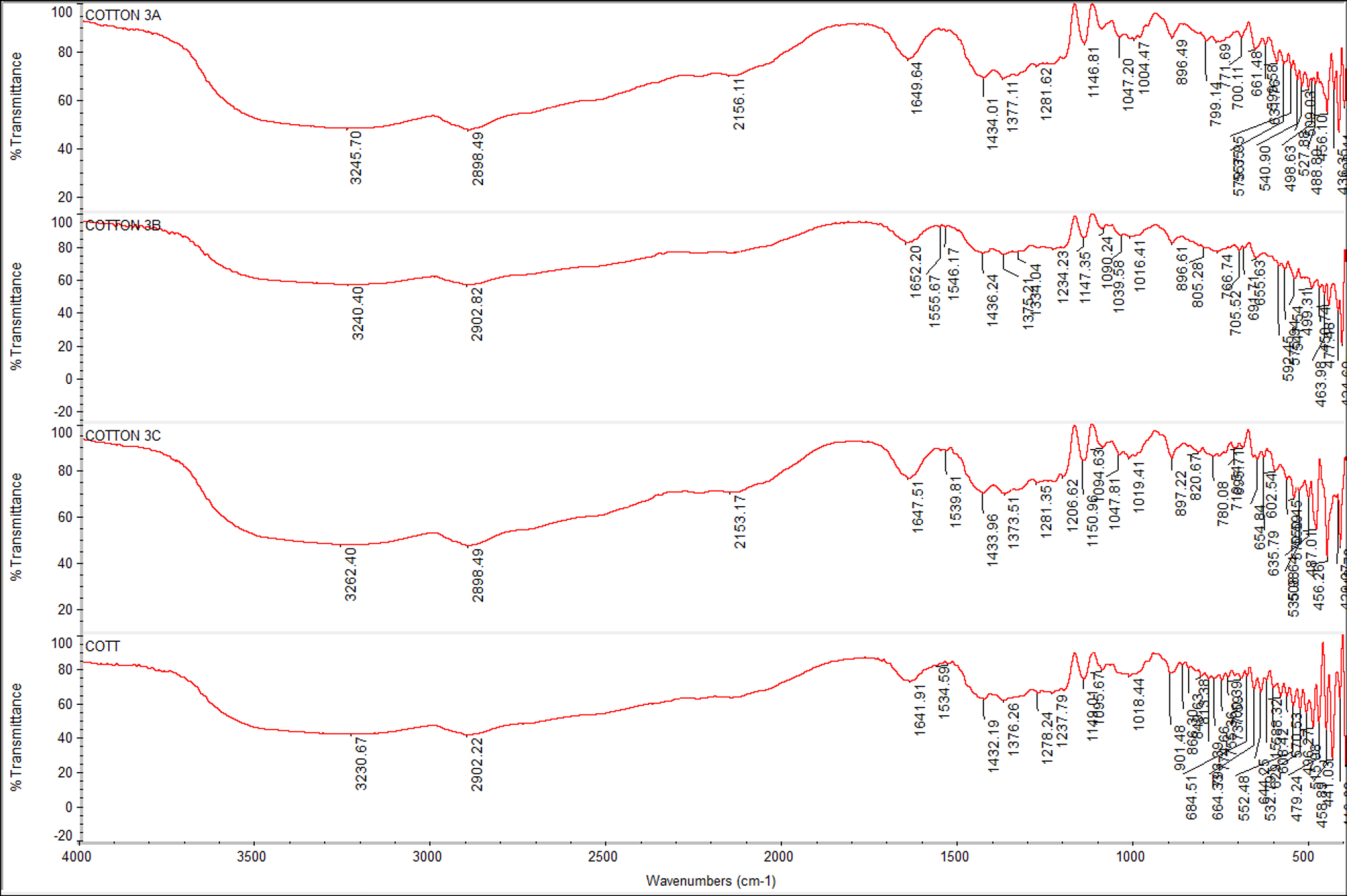
FTIR of undyed cotton fabric and dyed cotton fabric with dyes **3a**, **3b**, and **4**.

**Fig. 13:**
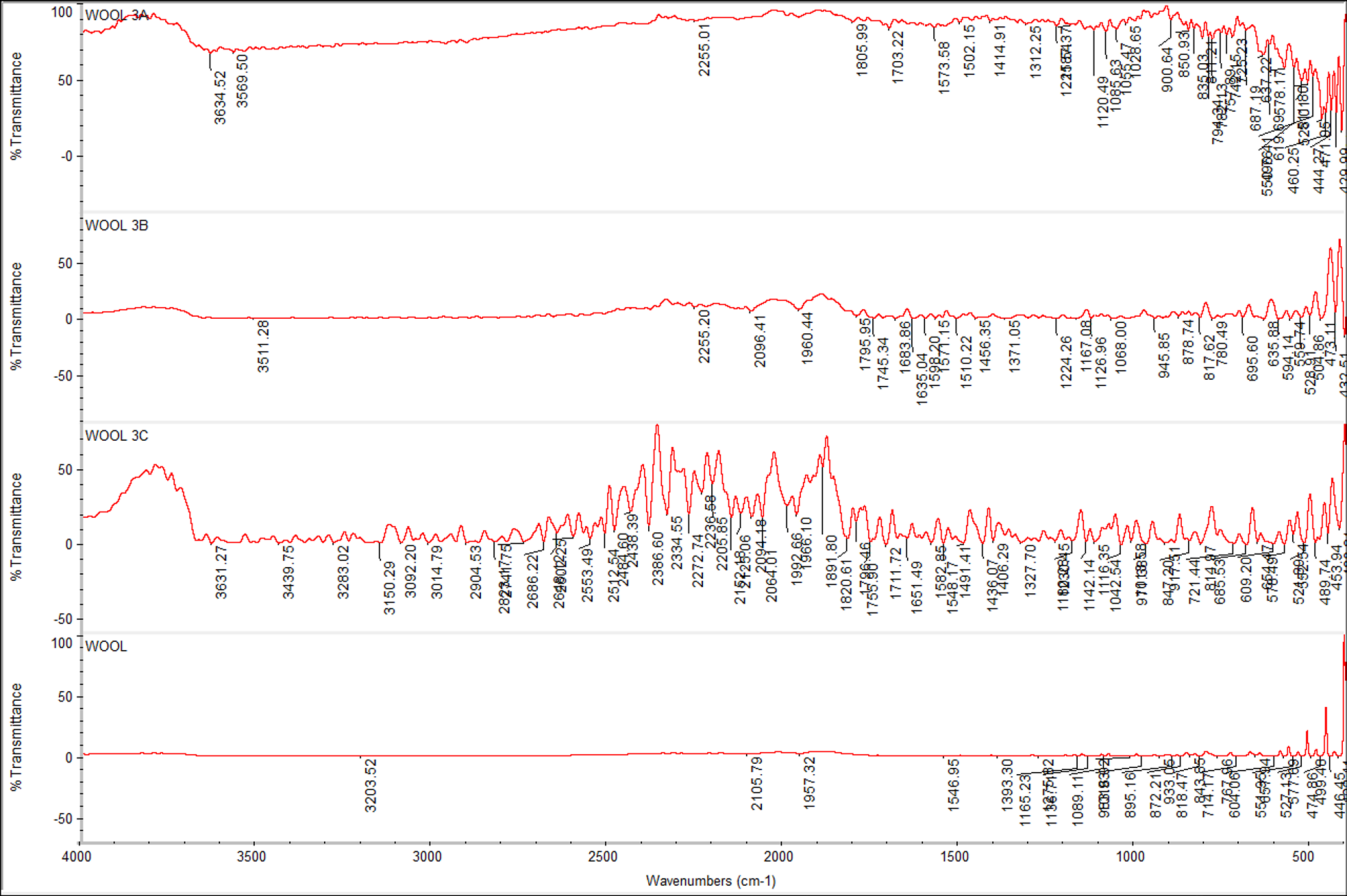
FTIR of undyed wool fabric and dyed wool fabric with dyes **3a**, **3b**, and **4**.

**Fig. 14:**
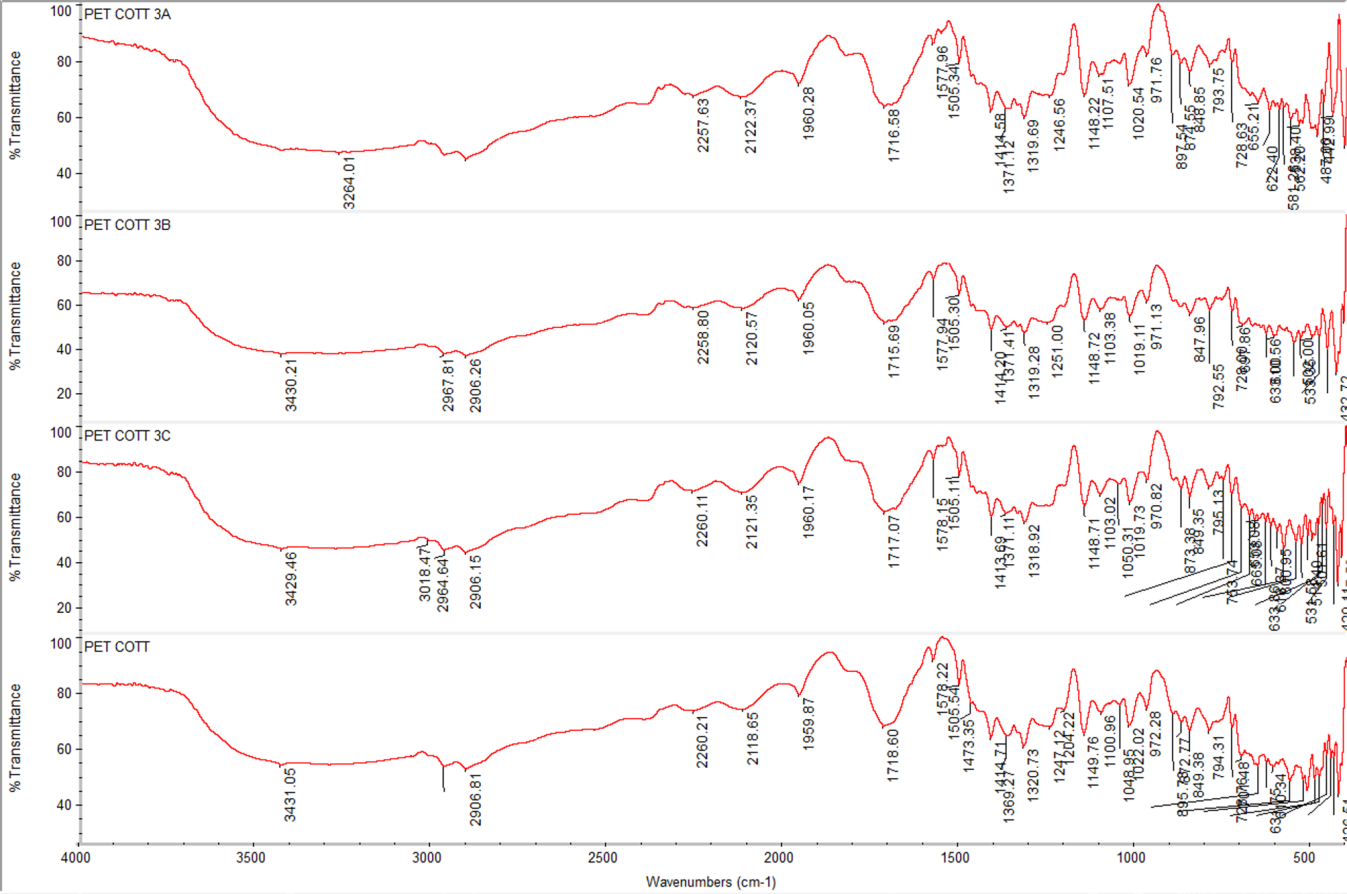
FTIR of undyed blend polyester/cotton fabric and dyed blend polyester /cotton fabric with dyes **3a**, **3b**, and **4**.

**Fig. 15:**
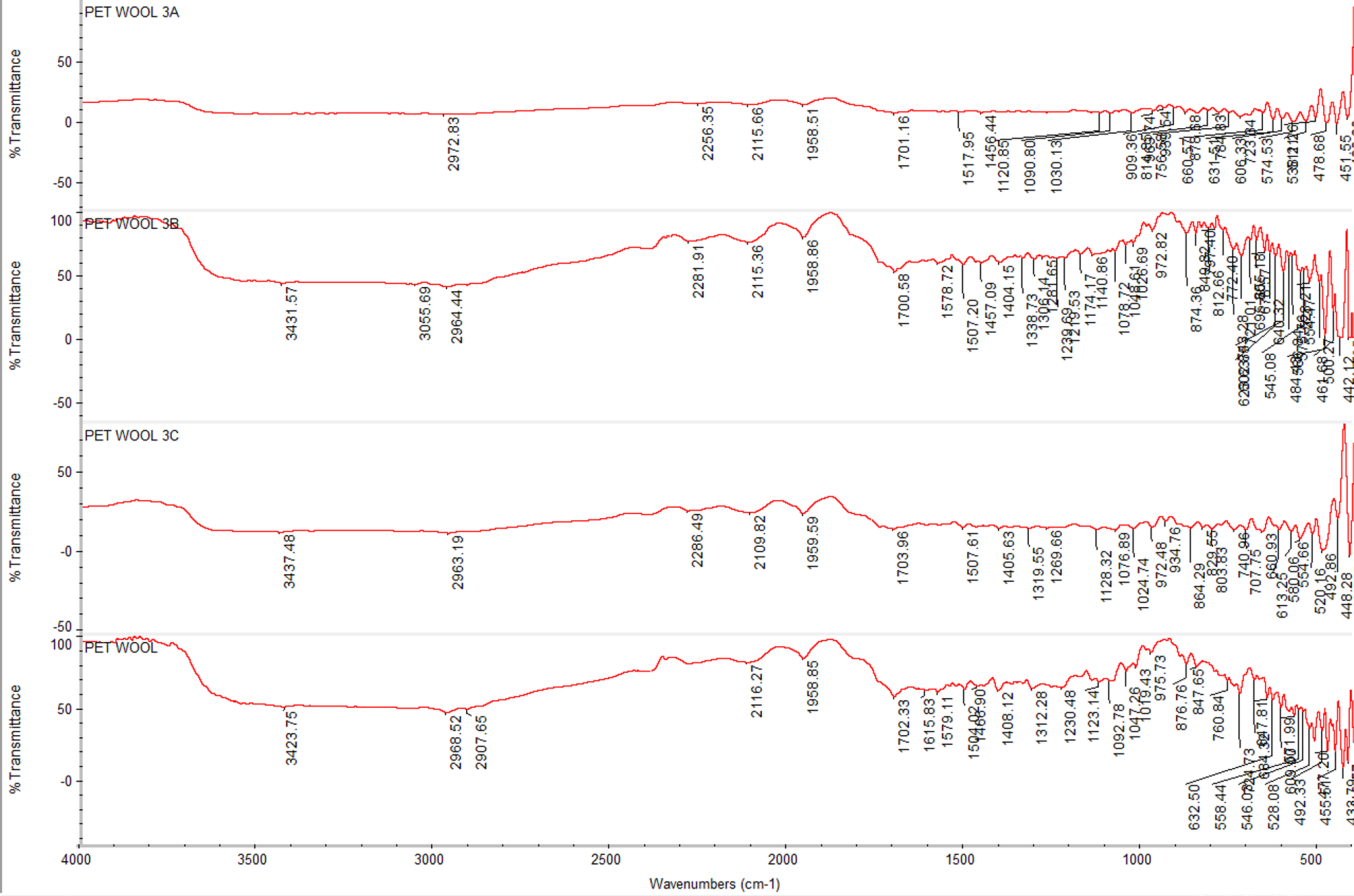
FTIR of undyed blend polyester/wool fabric and dyed blend polyester /wool fabric with dyes **3a**, **3b**, and **4**.

**Fig. 16:**
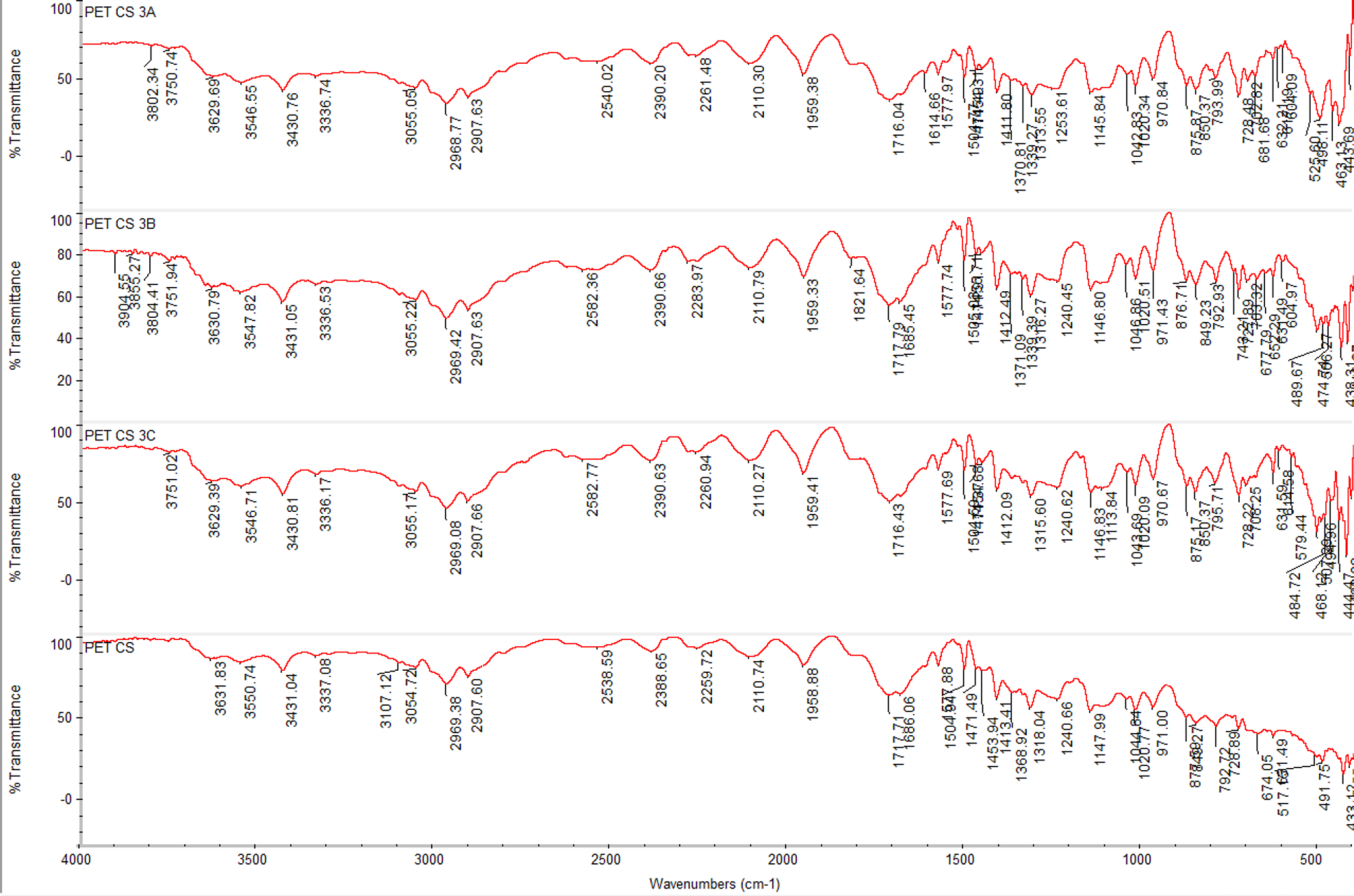
FTIR of undyed modified polyester/chitosan as a blend fabric and dyed modified polyester /chitosan as a blend fabric with dyes **3a**, **3b**, and **4**.

The fixation of dyestuff on pure, mixed, and modified fabrics was confirmed using Fourier-transform infrared spectroscopy (FTIR). In comparison to the undyed fabrics, the FTIR analysis of dyed and undyed fabrics (polyester, cotton, wool, polyester/cotton, polyester/wool, and polyester/chitosan) indicated the appearance of new peaks and a shift in intensity. This suggests that, in addition to physical bonding (hydrogen bonds, van der Waals forces) between synthetic dyes and functional groups of various textiles, there are interactions (covalent bonds) between reactive groups, particularly in dye **4**, which has two reactive centers. For instance, the intensity of most peaks was altered for polyester-dyed fabrics (Fig. 11); additionally, dyed cotton fabrics indicated an appearance of new peaks at 2156 and 2153 cm⁻¹ in addition to the changing intensity of nearly all peaks for dyes **3a** and **4** (Fig. 12). Furthermore, dyed wool fabrics showed three new peaks for dye **4** at 3631, 3439, and 3238 cm^−^¹; two new peaks for dye **3a** at 3634 and 3569 cm^−^¹; and one new peak for dye **3b** at 3511 cm^−^¹, in addition to the new peak for the dye’s characteristic functional groups (C = O) at 1703–1745 cm^−^¹, indicating a deeper interaction of dye **4** compared to the others (Figs. 13, 14, 15, 16).

### Antimicrobial profiling of the synthesized nano pyrazolone reactive dyes

**Fig. 17:**
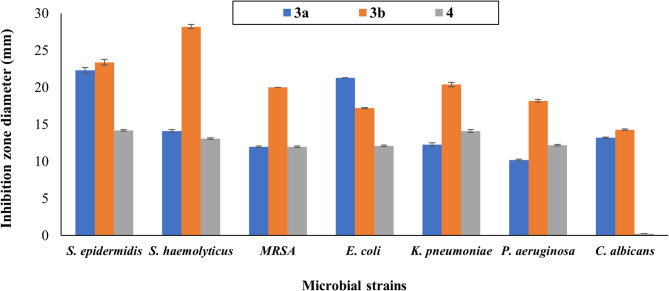
Antimicrobial activity of the prepared nano-disperse reactive dyes expressed as inhibition zone diameters (mm). Values are presented as mean ± SD (standard deviation) of three independent replicates (*n* = 3). DMSO was added to different organisms as a negative control and showed no inhibition zone.

**Fig. 18:**
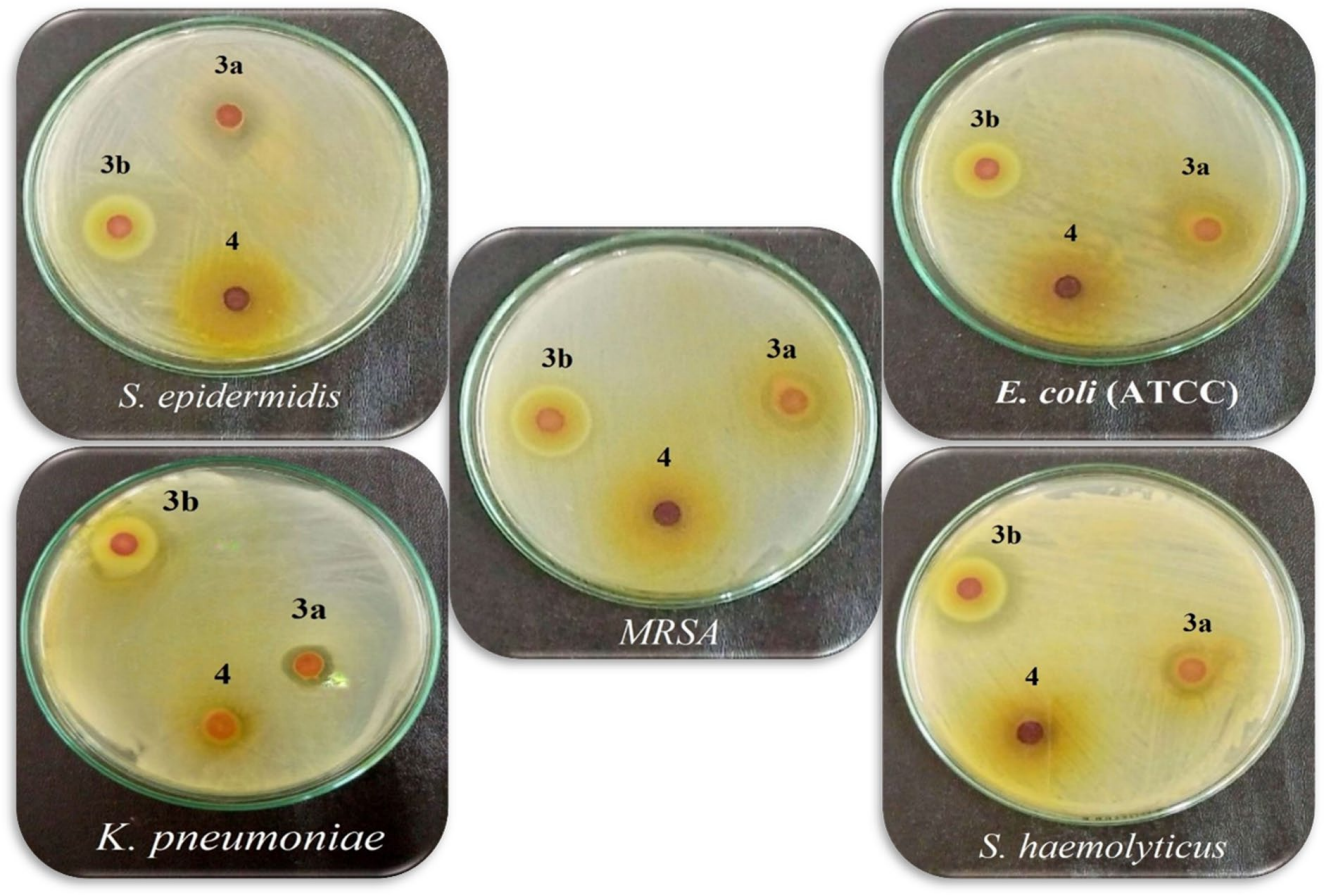
Some representative images of the antimicrobial activity of the synthesized nano-disperse reactive dyes.

The quantitative antimicrobial profiling, as delineated in (Figs. 17 and 18), revealed a compelling and structurally dependent spectrum of bioactivity for the synthesized nano-pyrazolone derivatives. The data underscore a pronounced dichotomy in efficacy, with compound **3b** emerging as the preeminent candidate, exhibiting a notably broad-spectrum and potent inhibitory profile. Compound **3b** demonstrates exceptional, broad-spectrum potency, particularly against clinically challenging pathogens. Its remarkable activity against *Staphylococcus haemolyticus* (28.2 mm) and *S. epidermidis* (23.4 mm) is the most outstanding feature, indicating a potentially unique or highly optimized interaction with this strain. Also, this compound achieved a potent inhibiting activity (17.2 mm) against the reference strain *E. coli* ATCC. Furthermore, its robust efficacy against methicillin-resistant *Staphylococcus aureus* (*MRSA*) (20.0 mm) and significant activity against the Gram-negative *Klebsiella pneumoniae* (20.4 mm) and *Pseudomonas aeruginosa* (18.2 mm) suggest a mechanism capable of overcoming common resistance barriers. The concurrent, measurable activity against *Candida albicans* (14.3 mm) nominates **3b** as a genuinely potent antimicrobial agent.

In contrast, compound **3a** presents a more selective spectrum, showing pronounced activity against *S. epidermidis* and *E. coli* (ATCC) (22.3 and 21.3 mm, respectively) but diminished effects against other bacterial strains, showing 14.1, 12.0, 12.3, and 10.2 mm zones of inhibition against *S. haemolyticus*,* MRSA*,* K. pneumoniae*, and *P. aeruginosa*, respectively. In addition, it had resulted in a 13.2 mm inhibition zone against the investigated pathogenic fungal strain *C. albicans*. Compound **4** exhibited the most constrained activity, with moderate inhibition zones against the investigated bacterial strains and a complete absence of antifungal activity against *C. albicans*, highlighting a significant attenuation of bioactivity relative to its counterparts. This compound achieved a moderate inhibition zone of 14.2, 13.1, 12.0, 12.1, 14.1, and 12.2 mm against *S. epidermidis*,* S. haemolyticus*,* MRSA*,* E. coli*,* K. pneumoniae*, and *P. aeruginosa*, respectively.

The stark differential in zone diameters, especially the superior and balanced performance of **3b**, illuminates a critical structure-activity relationship. This derivative’s consistent and potent cross-kingdom activity positions it as a singularly promising lead for advanced mechanistic studies and targeted antimicrobial development.

The antimicrobial evaluation of the synthesized nano-disperse reactive dyes 3a, 3b, and 4 revealed a compelling structure-activity relationship (SAR) that directly correlates molecular architecture with biological potency. This analysis underscores how subtle modifications to the pyrazolone core, specifically, the electronic nature of the aryl substituent and the number of reactive anchoring groups, profoundly influence the spectrum and efficacy of antimicrobial action. The pronounced bioactivity of these dyes can be attributed, in part, to their core pyrazolone heterocycle, a scaffold well-documented for its diverse pharmacological properties, including antimicrobial effects^25^. The mechanism likely involves interactions with microbial cell walls and membranes, potentially leading to disruption of integrity and function. The nano-scale size of the dye particles, achieved via ball milling, significantly enhances this interaction by providing a high surface area-to-volume ratio, facilitating greater contact with and penetration into microbial cells, a phenomenon supported by recent studies on nanostructured antimicrobial agents^20^.

The SAR is most strikingly illustrated by the superior performance of compound **3b**. This derivative, featuring an electron-donating amino group (-NH_2_) on the phenyl ring attached to the pyrazolone, emerged as the most potent broad-spectrum agent. The -NH_2_ group is known to enhance solubility and facilitate hydrogen bonding and electrostatic interactions with negatively charged components of microbial membranes, such as lipopolysaccharides in Gram-negative bacteria and teichoic acids in Gram-positive bacteria^20^. This explains the exceptional activity of **3b** against both cohorts, including the formidable MRSA and multidrug-resistant *S. haemolyticus*. Its concurrent activity against *C. albicans* suggests an action mechanism that may also disrupt fungal ergosterol synthesis or cell wall β-glucans, a promising feature for combating fungal infections^24^.

In contrast, compound **3a**, which bears a strong electron-withdrawing nitro group (-NO_2_), displayed a more selective antimicrobial profile. While it maintained good activity against *S. epidermidis* and *E. coli*, its potency against other strains was markedly diminished. The -NO_2_ group reduces electron density on the aromatic system, potentially decreasing the ability of the compound to engage in the crucial polar interactions necessary for broad-spectrum activity. This aligns with findings that electron-withdrawing substituents can attenuate the membrane-disrupting potential of antimicrobial agents by making them less likely to interact with anionic phospholipid head groups^26^. The most revealing aspect of the SAR is the significant attenuation of bioactivity observed in compound **4**. Despite possessing two reactive centers (vinyl sulfone and cyanuric chloride), its antimicrobial potency was the lowest, and it lacked antifungal action entirely. The introduction of the bulky, electrophilic cyanuric chloride moiety likely alters the molecule’s electronic distribution, steric profile, and possibly its mode of interaction with microbial targets. The molecule may become more predisposed to covalent bonding with fabric nucleophiles than with microbial components, or its increased hydrophilicity/hydrophobicity balance may hinder membrane penetration^40^. This finding is pivotal, demonstrating that optimizing a dye for covalent fixation on textiles does not inherently translate to enhanced antimicrobial performance and may even be detrimental.

All in all, the antimicrobial data delineate a clear SAR: an electron-donating group (e.g., -NH_2_) on the pyrazolone aryl ring is conducive to broad-spectrum, potent antimicrobial activity, while electron-withdrawing groups (e.g., -NO_2_) narrow the spectrum. Furthermore, the addition of a second, bulky reactive group for textile fixation can significantly compromise antimicrobial potency, highlighting a functional trade-off. Compound **3b** stands out as an exemplary lead, successfully balancing potent, cross-kingdom antimicrobial action with effective dyeing properties. Future work should focus on elucidating the precise mechanism of action of **3b**, possibly involving membrane depolarization assays or intracellular target identification^41^, and evaluating the durability of its antimicrobial activity after fixation on various fabrics under a standardized wash testing protocol.

### Antimicrobial efficacy by colony-forming unit (CFU) assay

**Fig. 19:**
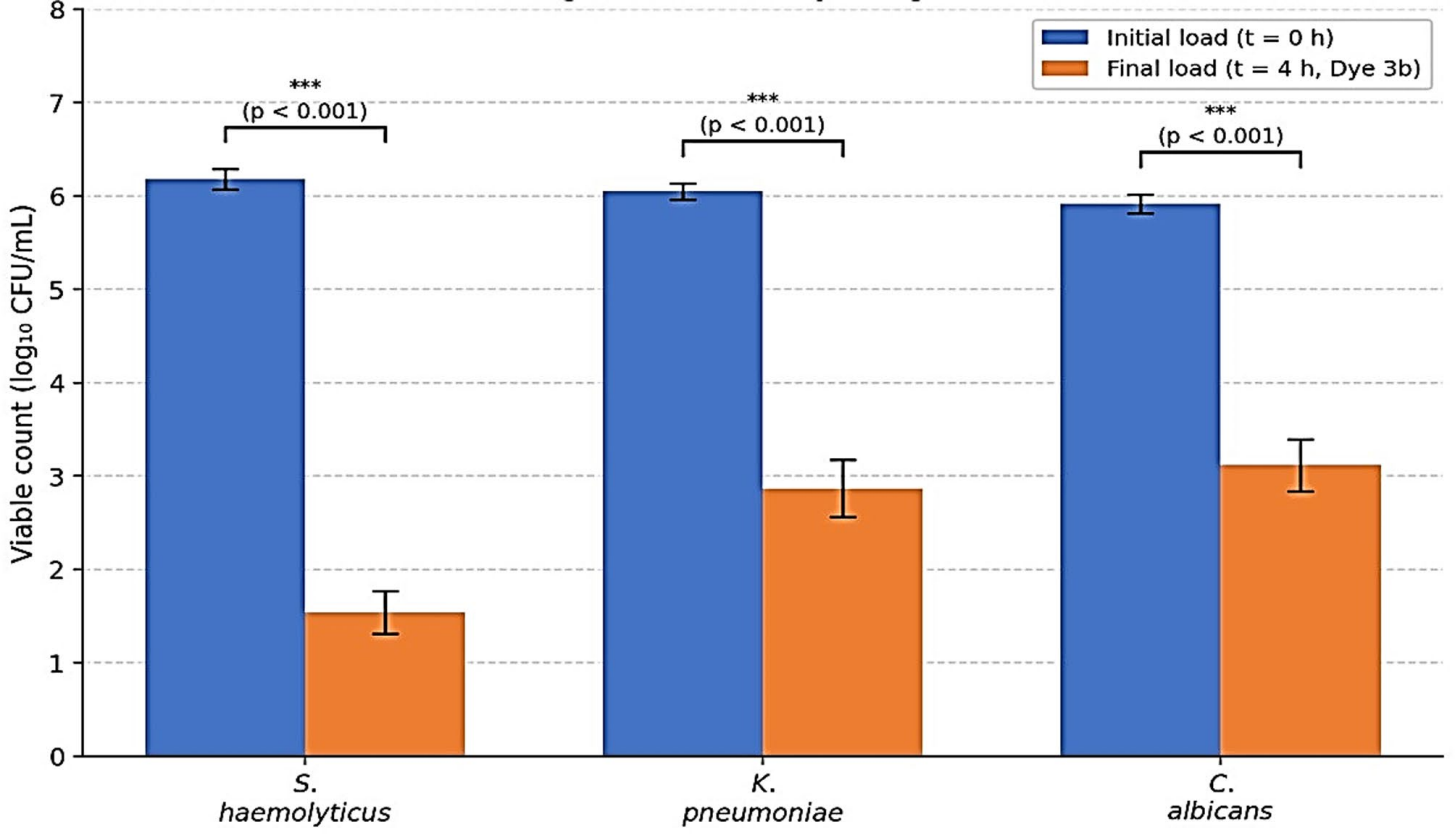
Quantitative CFU assay showing the antimicrobial efficacy of dye **3b** against selected pathogens. Error bars represent ± SD of three independent replicates (*n* = 3). ***p < 0.001 vs. initial load (one-way ANOVA followed by Tukey’s post-hoc test). The DMSO negative control showed no significant reduction compared to the initial load.

As shown in Fig. 19, compound 3b exhibited a dramatic reduction in viable counts against all three tested pathogens. Against *S. haemolyticus*, the viable count decreased from an initial load of 6.18 log_10_ CFU/mL to 1.54 log_10_ CFU/mL after 4 h of treatment, representing a reduction of approximately 4.64 log_10_ (99.998%). Against *K. pneumoniae*, the count dropped from 6.05 log_10_ CFU/mL to 2.87 log_10_ CFU/mL (reduction of 3.18 log_10_; 99.93%). Against *C. albicans*, the viable count decreased from 5.92 log_10_ CFU/mL to 3.12 log_10_ CFU/mL (reduction of 2.80 log_10_; 99.84%). The negative control (DMSO) showed no significant reduction in viable counts compared to the initial load. These quantitative CFU data confirm the potent and broad-spectrum antimicrobial activity of dye 3b, particularly its remarkable bactericidal effect against the multidrug-resistant Gram-positive pathogen *S. haemolyticus*, supporting its potential for health-related textile applications.

The potent antimicrobial activity of dye 3b is attributed to its nano-scale particle size (7.88 nm as confirmed by TEM), which provides an exceptionally high surface area-to-volume ratio, maximizing electrostatic interaction with negatively charged microbial surfaces. The electron-donating amino group (–NH_2_) in dye 3b enhances its ability to form hydrogen bonds and electrostatic attractions with anionic components of the bacterial cell envelope, including lipopolysaccharides (LPS) in Gram-negative bacteria and teichoic acids in Gram-positive bacteria^20^. Furthermore, the nano-dye particles can penetrate the microbial cell wall, cause the disruption of membrane integrity and lead to leakage of intracellular contents^21^. Recent studies have confirmed that pyrazole-based nano-compounds induce membrane depolarization and inhibit biofilm formation, which aligns with the observed log reductions in CFU assays^24,25^. The remarkable 4.64 log_10_ reduction against multidrug-resistant *S. haemolyticus* indicates a bactericidal rather than bacteriostatic mechanism, likely involving disruption of the phospholipid bilayer and interference with essential membrane-bound enzymes.

## Conclusion

In this research, three kinds of new nano-disperse reactive dyes based on the pyrazolone moiety have been synthesized by an eco-friendly method. One of these dyes has an electron-withdrawing group (EWG) (NO_2_) and one reactive group (vinyl sulfone) **3a**, while **3b** has an electron-donating group (EDG) (NH_2_) and one reactive group (vinyl sulfone), and the last one has two reactive groups (vinyl sulfone and cyanuric chloride) **4**, and they tested them on dyeing various fabrics (pure, blended, and modified) by traditional conditions to show how changing the substituent and reactive groups affected the efficiency of disperse reactive dyes on different materials. We found that **3a** with an electron-withdrawing group (EWG) has a higher K/S% value than **3b** with an electron-donating group (EDG), whereas dye **4** with two reactive centers has a higher K/S% value than **3b**, which has one reactive center. On the other hand, we tried to change the primary parameters, such as temperature, pH, time duration, and the use of auxiliaries in the dyeing process, by measuring the K/S% in this case, to achieve the optimal conditions to give the best affinity in the penetration of dyes onto fabrics. Beyond color properties, the dyes exhibited significant and structurally dependent antimicrobial activity against a panel of drug-resistant pathogens. Dye **3b** emerged as a potent broad-spectrum agent, showing exceptional activity against multidrug-resistant Gram-positive bacteria like *Staphylococcus haemolyticus* and MRSA, as well as considerable efficacy against Gram-negative bacteria (*K. pneumoniae*,* P. aeruginosa*) and the pathogenic yeast *C. albicans*. Dye **3a** displayed strong but more selective antibacterial activity, while dye **4** showed moderate antibacterial effects and no antifungal activity. Collectively, this work demonstrated that molecular design, specifically the choice of substituent and number of reactive groups, critically influences both the dyeing performance and the antimicrobial potency of these multifunctional nano-dyes. With this detailed study, we hope that the conditions described in the research will be applied to the dyeing of different types of fabrics soon. Our findings result in increased dye exhaustion and fixation rates, which in turn reduce the amount of dye remaining in wastewater, making the dyes used more environmentally friendly.

## Supplementary Information

Below is the link to the electronic supplementary material.

Supplementary Material 1

Supplementary Material 2

Supplementary Material 3

Supplementary Material 4

## Data Availability

All data generated or analyzed during this study are included in this published article.
