# Supplementary material for: Comparative studies targeting the best dyeing conditions for pure, blend and modified fabrics with novel nano-disperse reactive dyes and their biological activity
Source: Sci Rep. 2026 Jun 4;16:17374. doi: 10.1038/s41598-026-55597-4 (PMC13237350; doi:10.1038/s41598-026-55597-4)

**Supplementary Materials**

**Comparative studies targeting the best dyeing conditions for pure, blend and modified fabrics with novel nano disperse reactive dyes and their biological activity**

**Hala F. Rizk^a^, Hamada M. Mashaly^b^, Hamada S. A. Mandour^a,d^, Khalil M. Saad-Allah^c^, Mohamed AB. Mohamed^a^, Mohamed R. Sadek^a^, Amira K. Fares^a^**

*^a^ Department of Chemistry, Faculty of Science, Tanta University, Tanta 31527, Egypt*

*^b^ Dyeing and Printing Department, Textile Research and Technology Institute, National Research Center, Giza, 12622, Egypt*

*^c^ Department of Botany, Faculty of Science, Tanta University, Tanta 31527, Egypt*

*^d^ Alsalam University, Egypt*

*E-mail: (H.R.): [dr_hala_fawzy@yahoo.com](mailto:dr_hala_fawzy@yahoo.com); (ORCID: 0000-0001-5517-3935)

1. **Experiamental**

**Chemistry**

Acquired from Sigma-Aldrich, all analytical-grade chemicals, in addition to pyrazolone derivatives **1a** and **1b,** were utilized without any additional purification. Without any adjustments, all melting points were measured using Gallen kamp melting point equipment. The infrared spectra were recorded using a Perkin-Elmer FTIR 1430 spectrophotometer, with the KBr disk technique implemented for the measurement process, FTIR for fabrics AKX1200130, type Nicopet_1_is10. At 25^°^C in DMSO-*d_6_*_,_ the ^1^H and ^13^C NMR spectra were recorded using a Bruker AC spectrometer (500 MHz for ^1^H and 75 MHZ for ^13^C). Chemical shifts are reported in ppm as *δ* values, and the reported shifts were measured against an internal standard called TMS. At Al-Azhar University, mass spectra were recorded at 70 eV using a Finnigan MAT 8222 EX mass spectrometer. Thin layer chromatography (TLC), with petroleum ether/ethyl acetate (3/2 by volume) as the eluent, was employed to monitor the reaction's progress.

**Mass spectra of the synthesized compounds**

**Mass spectrum of compound 3a**


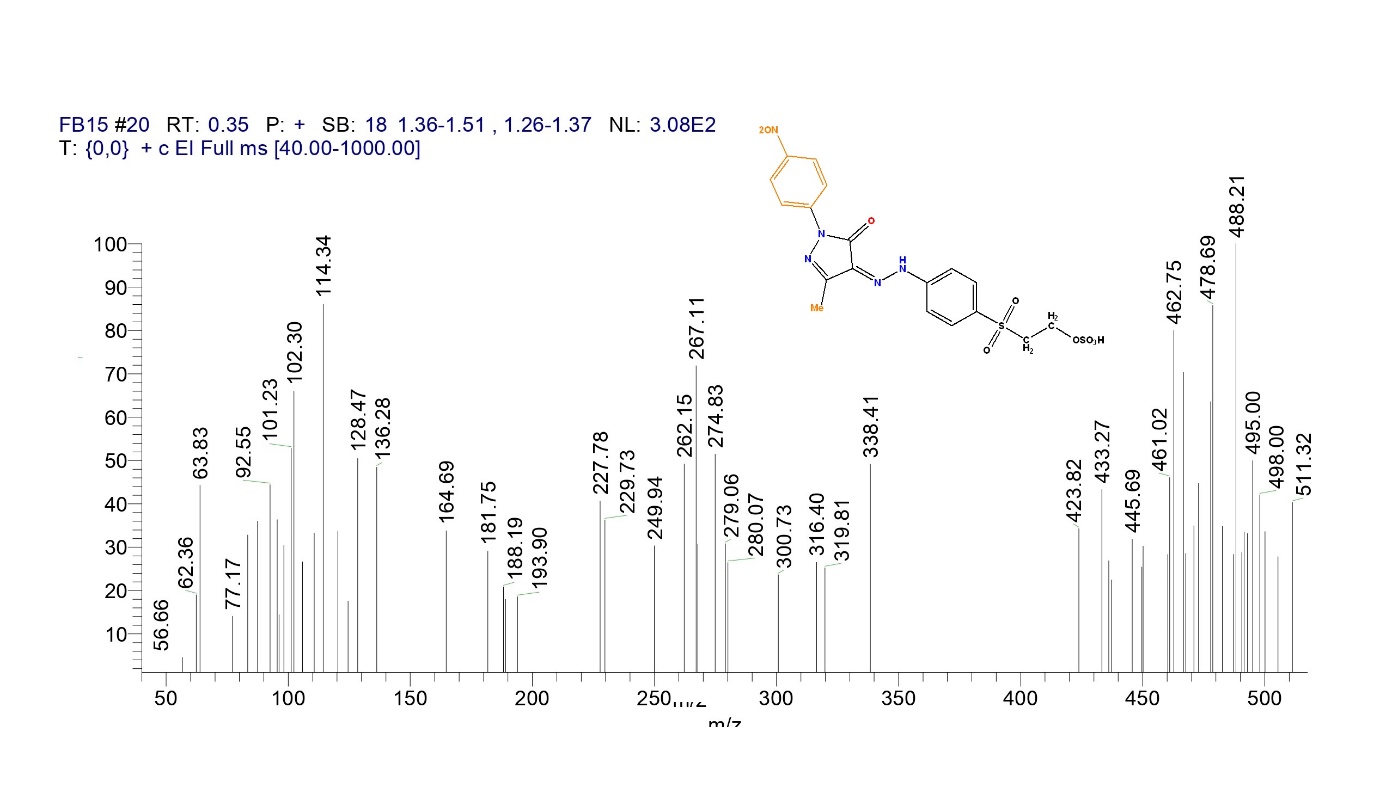


**Mass spectrum of compound 3b**


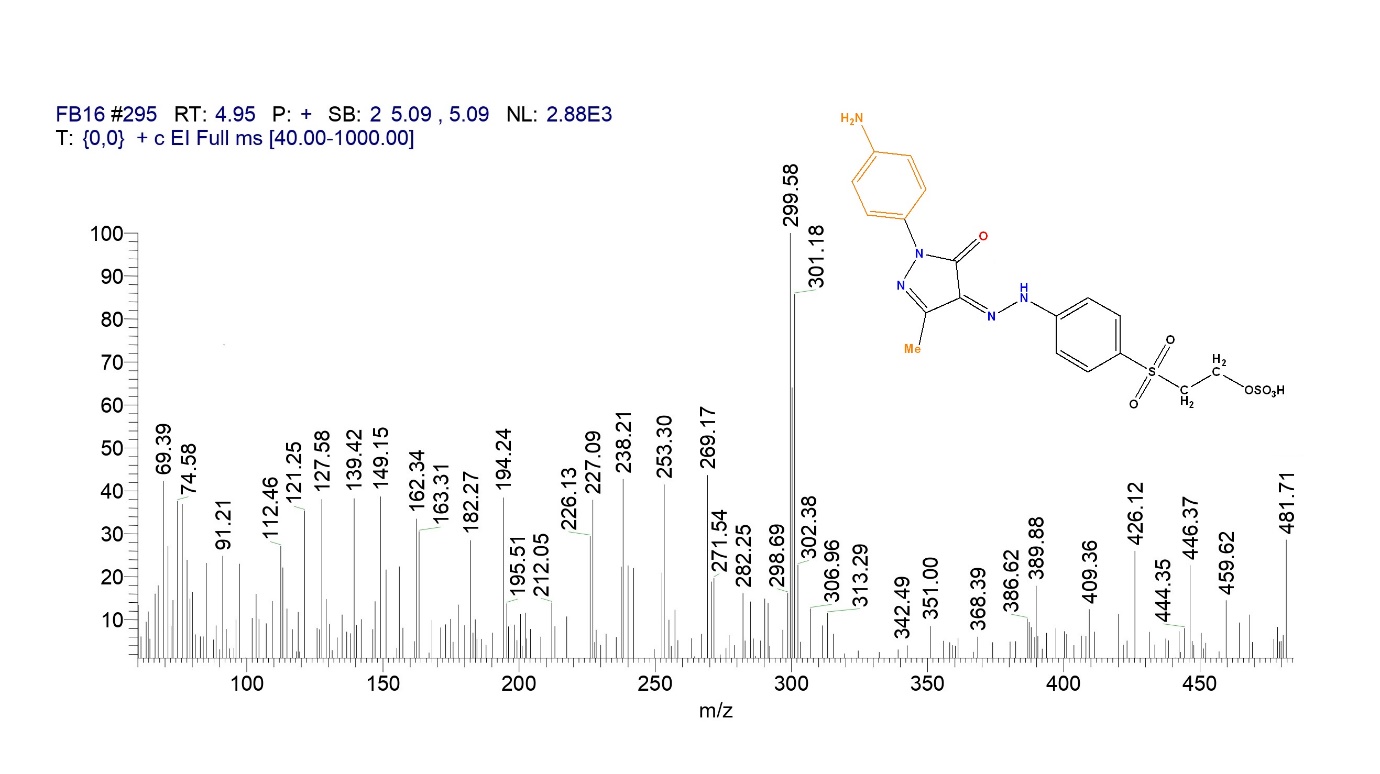


**Mass spectrum of compound 4**


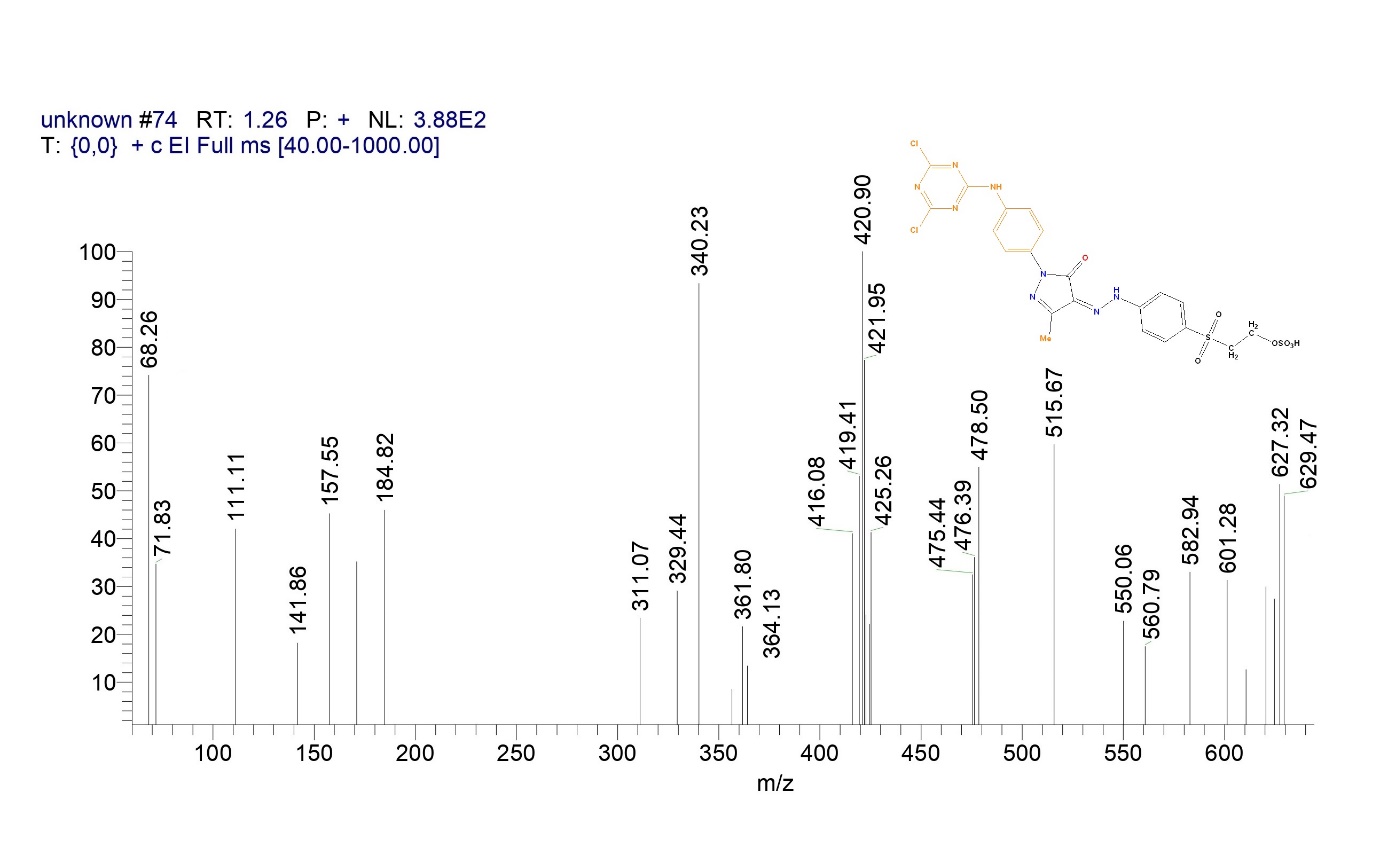


**^1^H NMR spectrum of compound 3a (DMSO)**


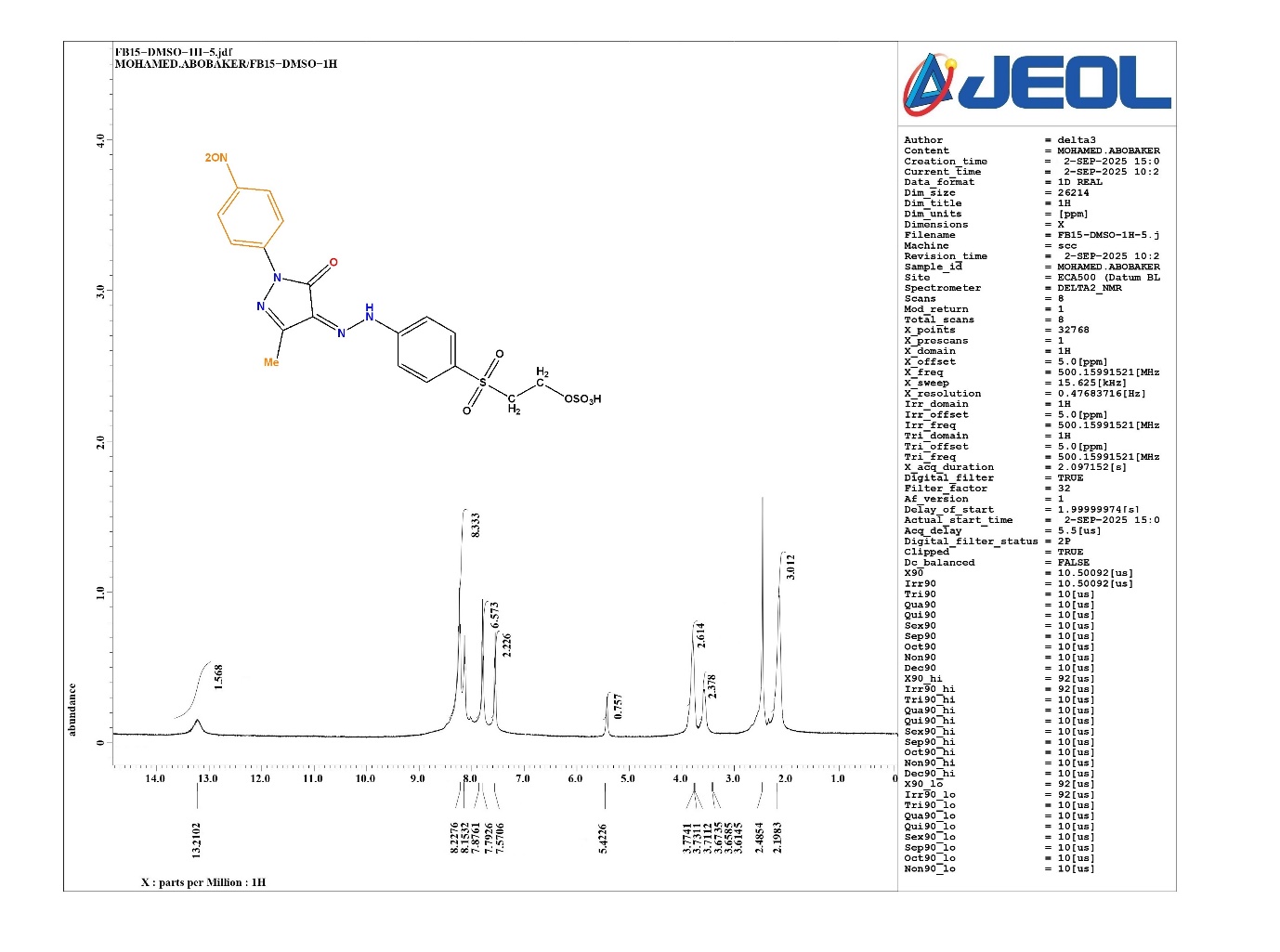


**^1^H NMR spectrum of compound 3a (DMSO-D_2_O)**


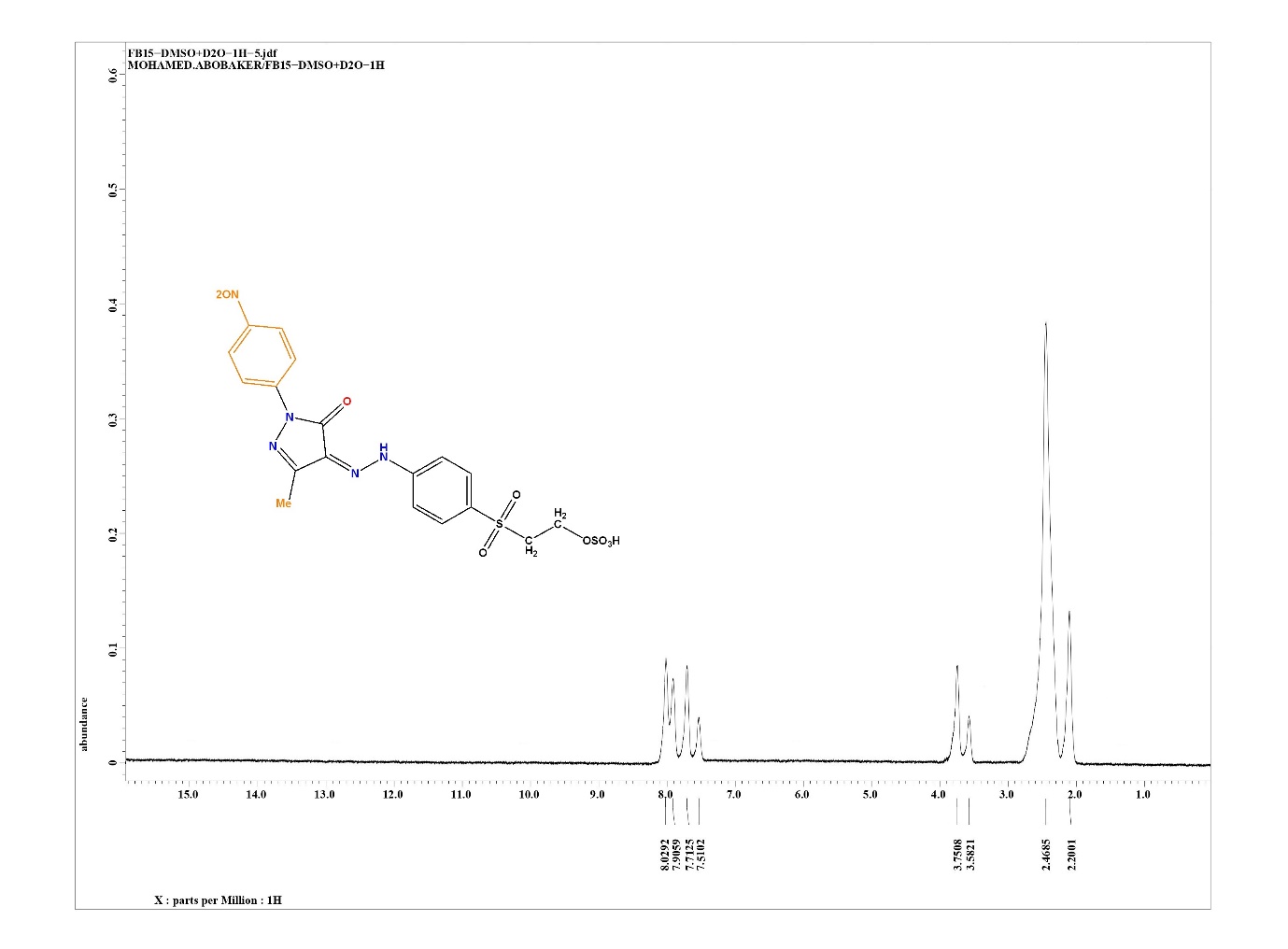


**^13^C NMR spectrum of compound 3a (DMSO)**


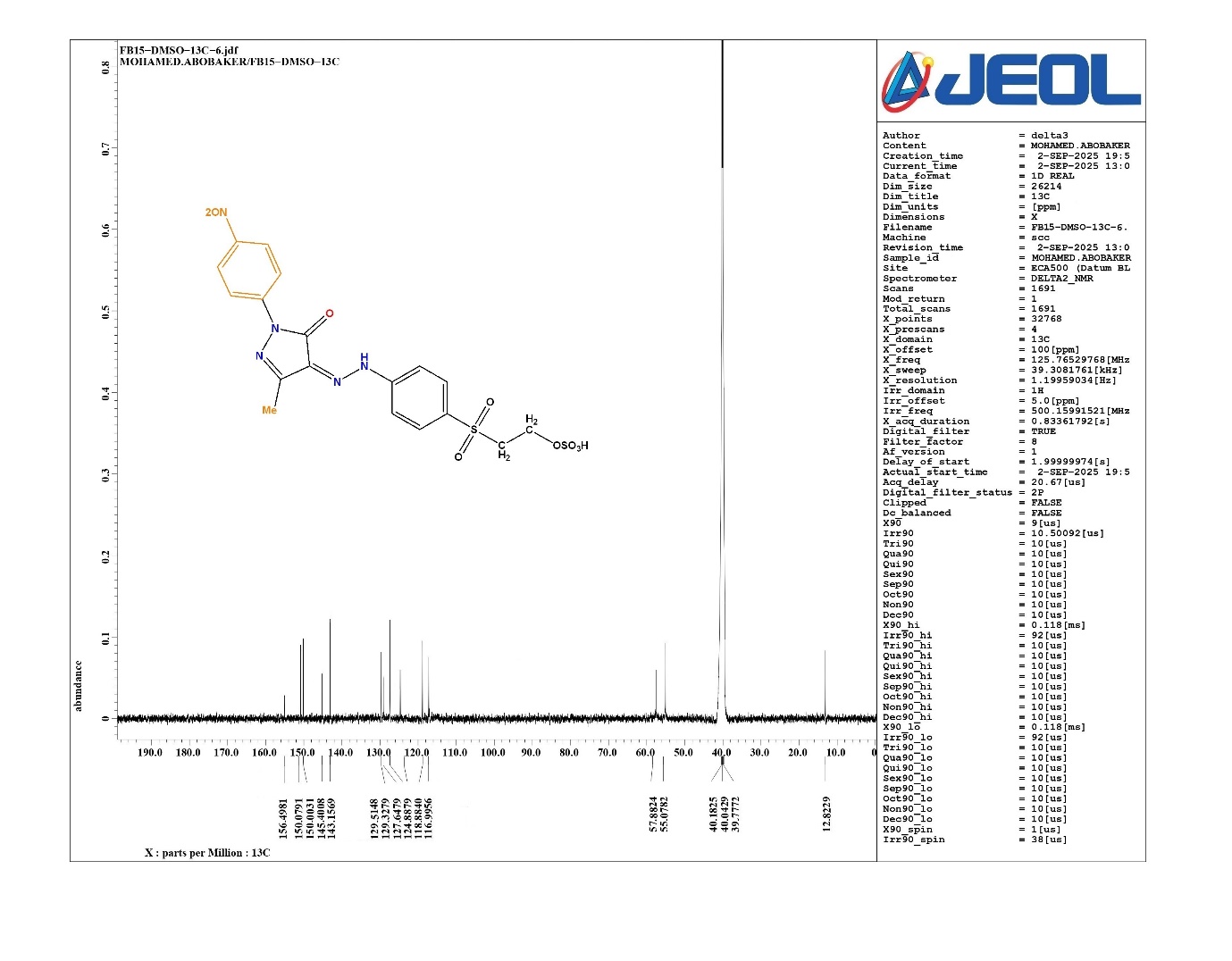


**^1^H NMR spectrum of compound 3b (DMSO)**


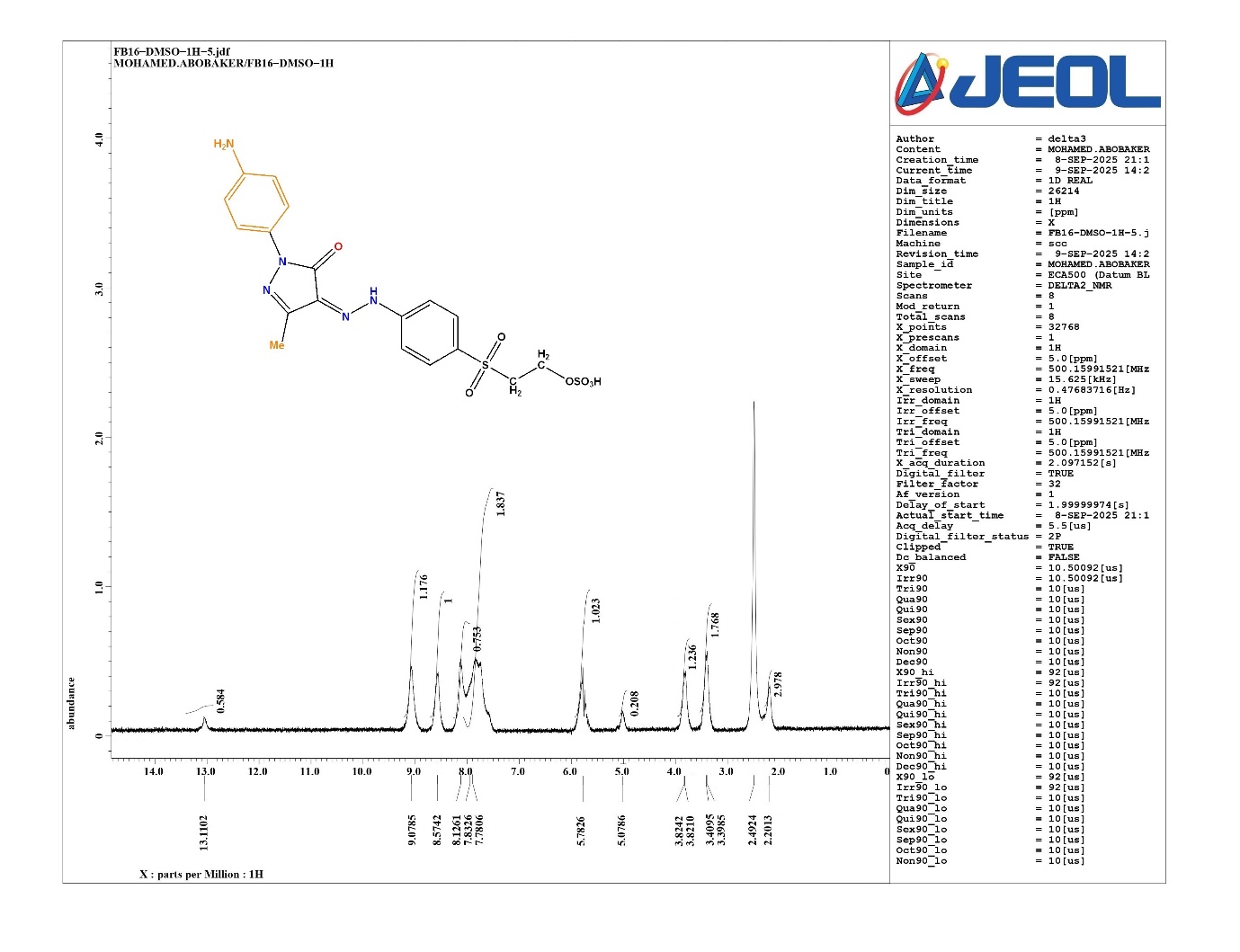


**^1^H NMR spectrum of compound 3b (DMSO-D_2_O)**


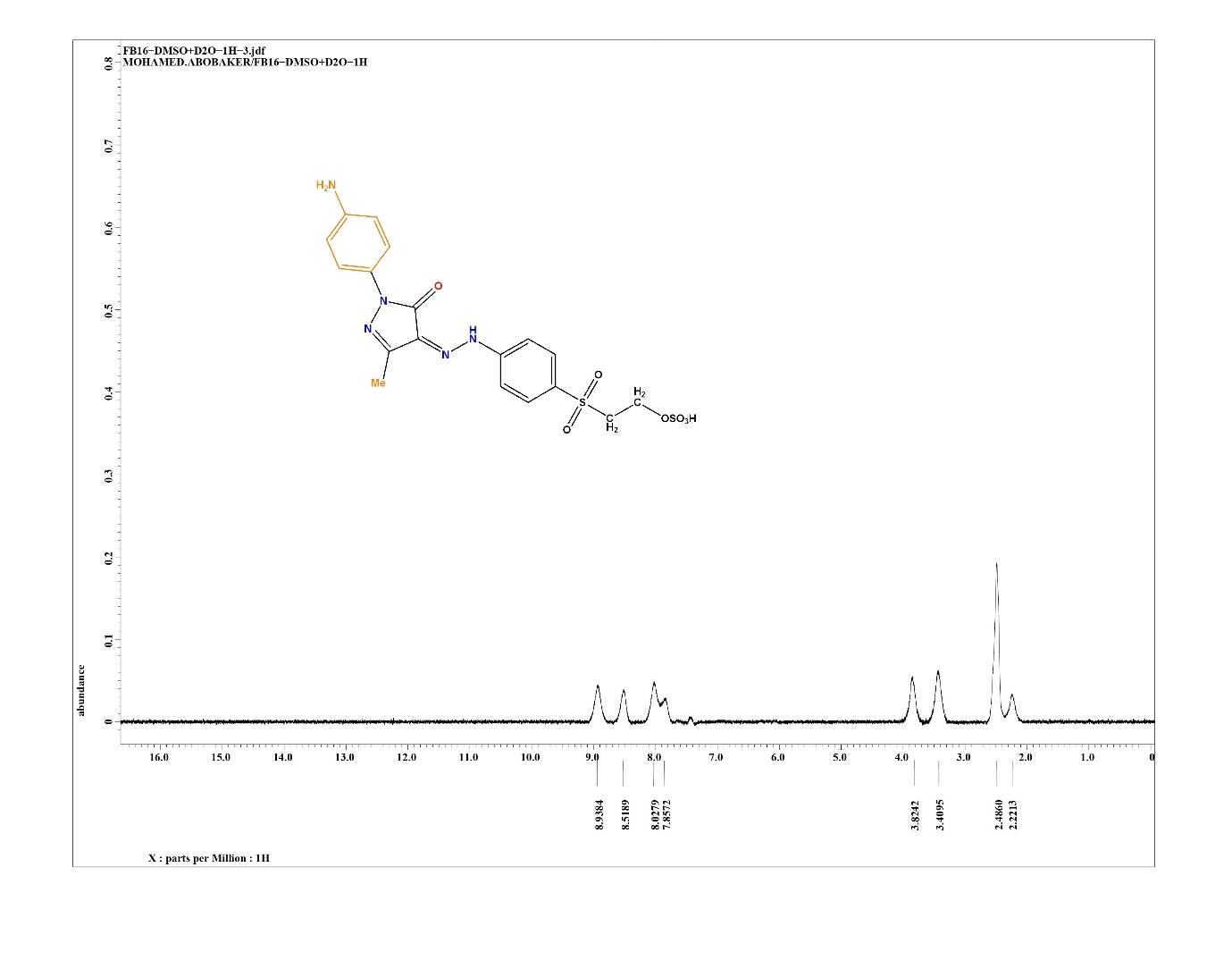


**^13^C NMR spectrum of compound 3b (DMSO)**


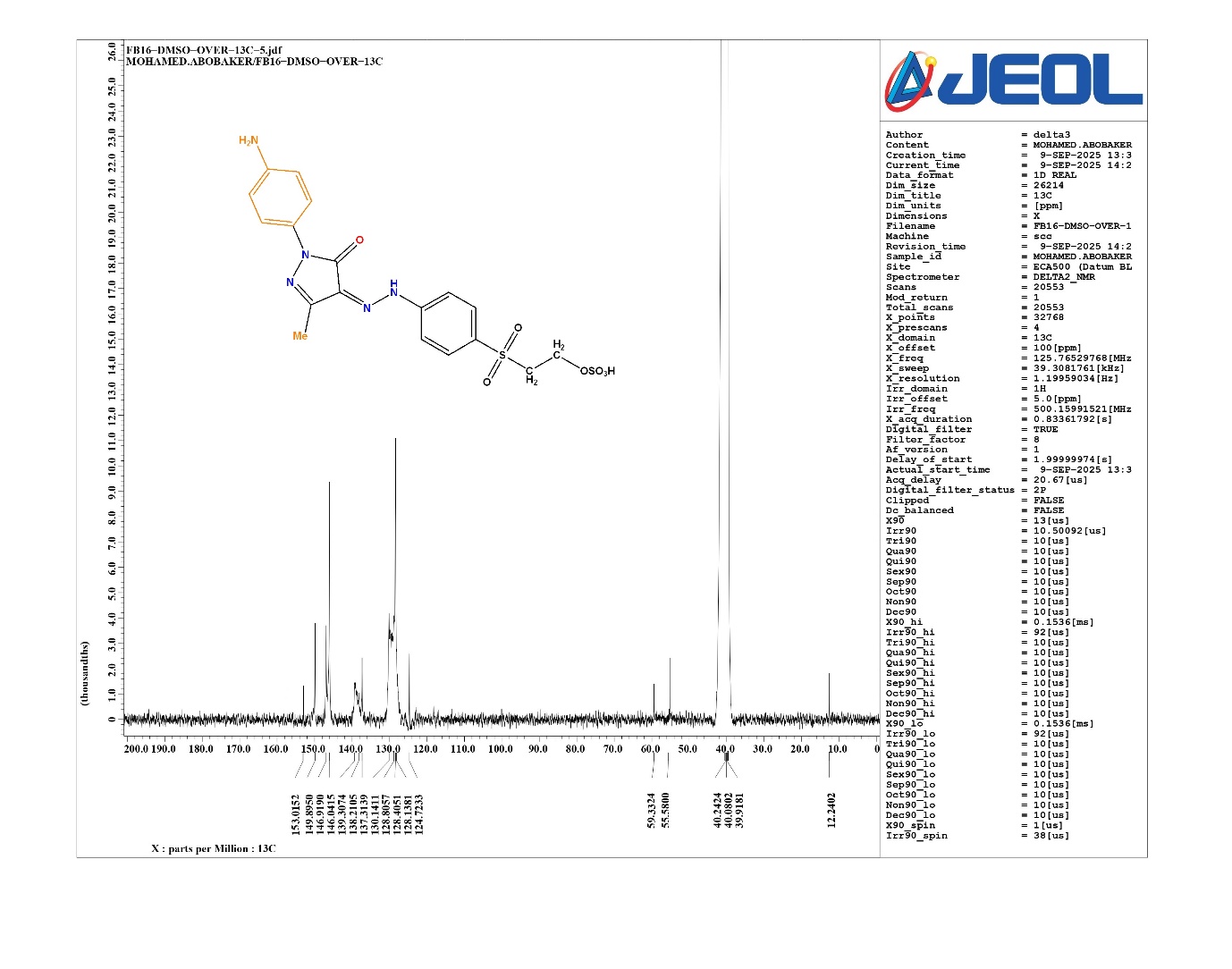


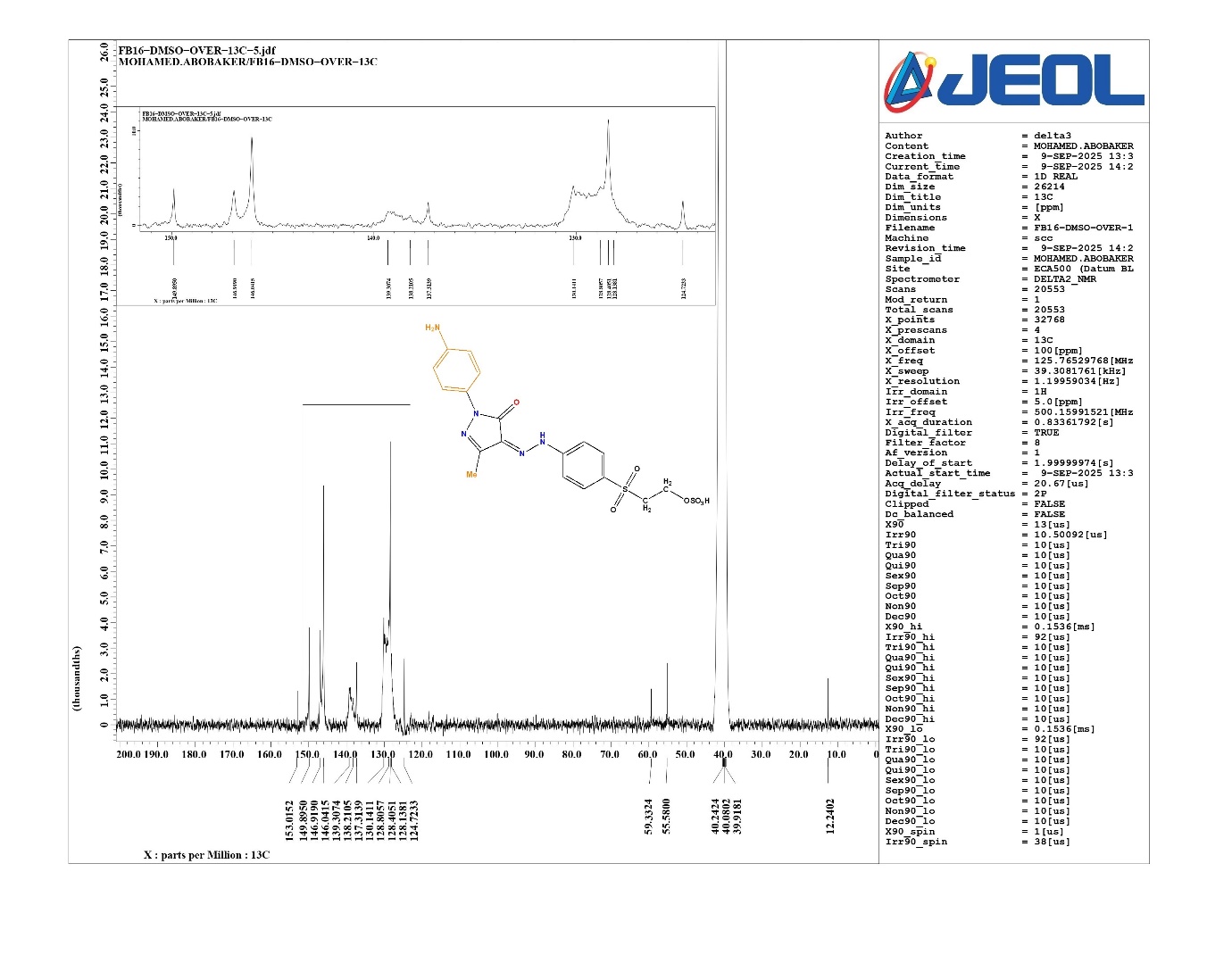


**^1^H NMR spectrum of compound 4 (DMSO)**


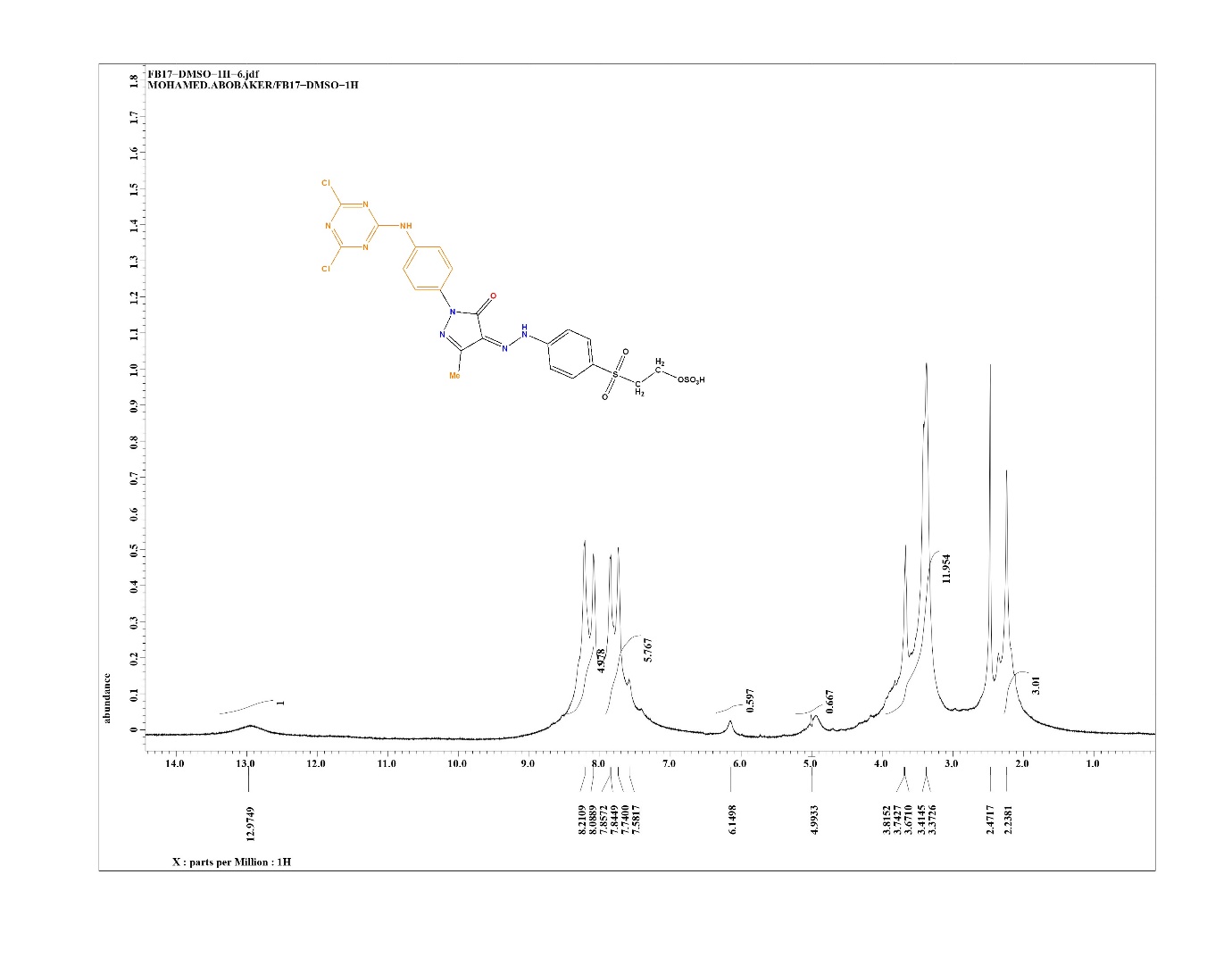


**^1^H NMR spectrum of compound 4 (DMSO-D_2_O)**


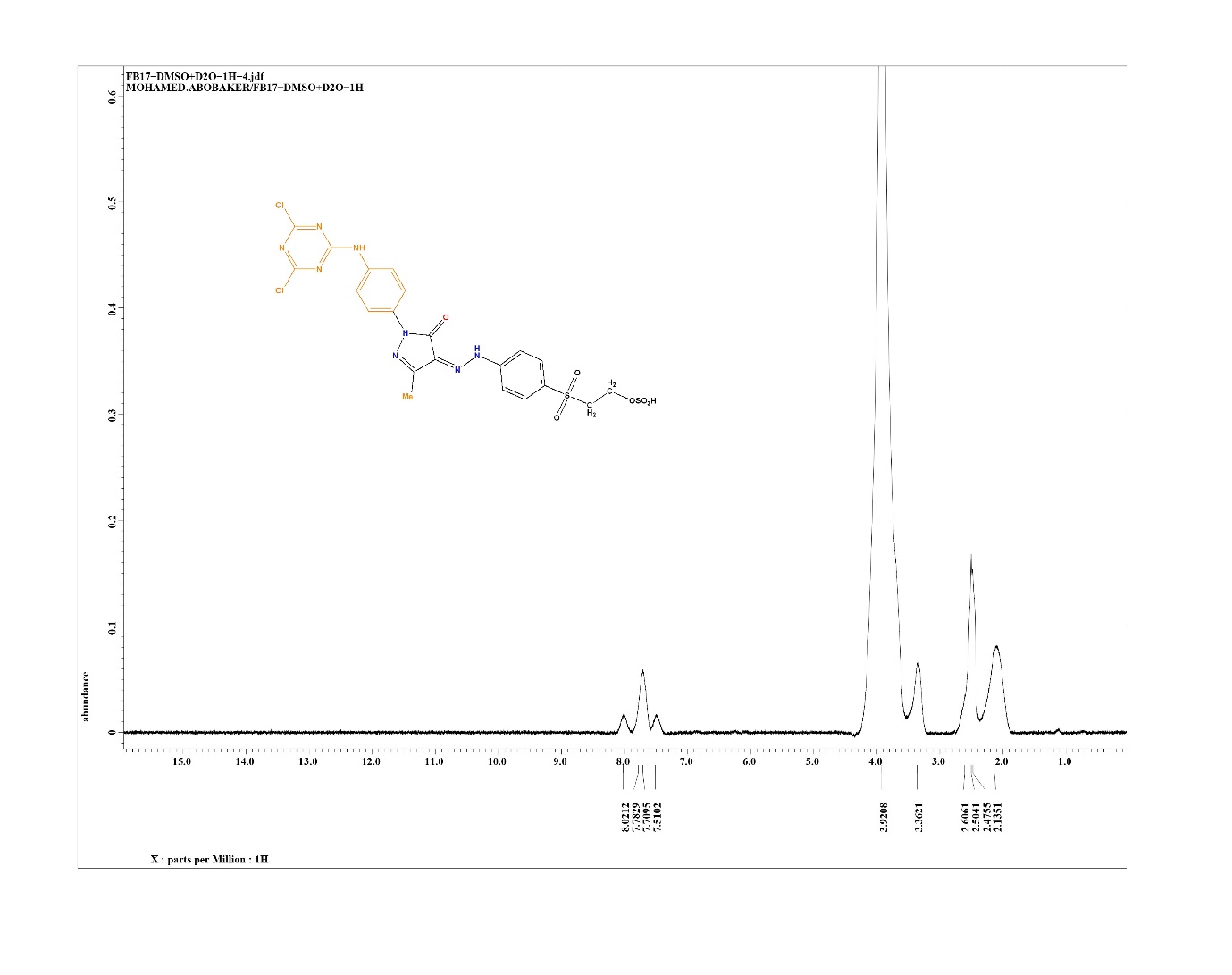


**^13^C NMR spectrum of compound 4 (DMSO)**


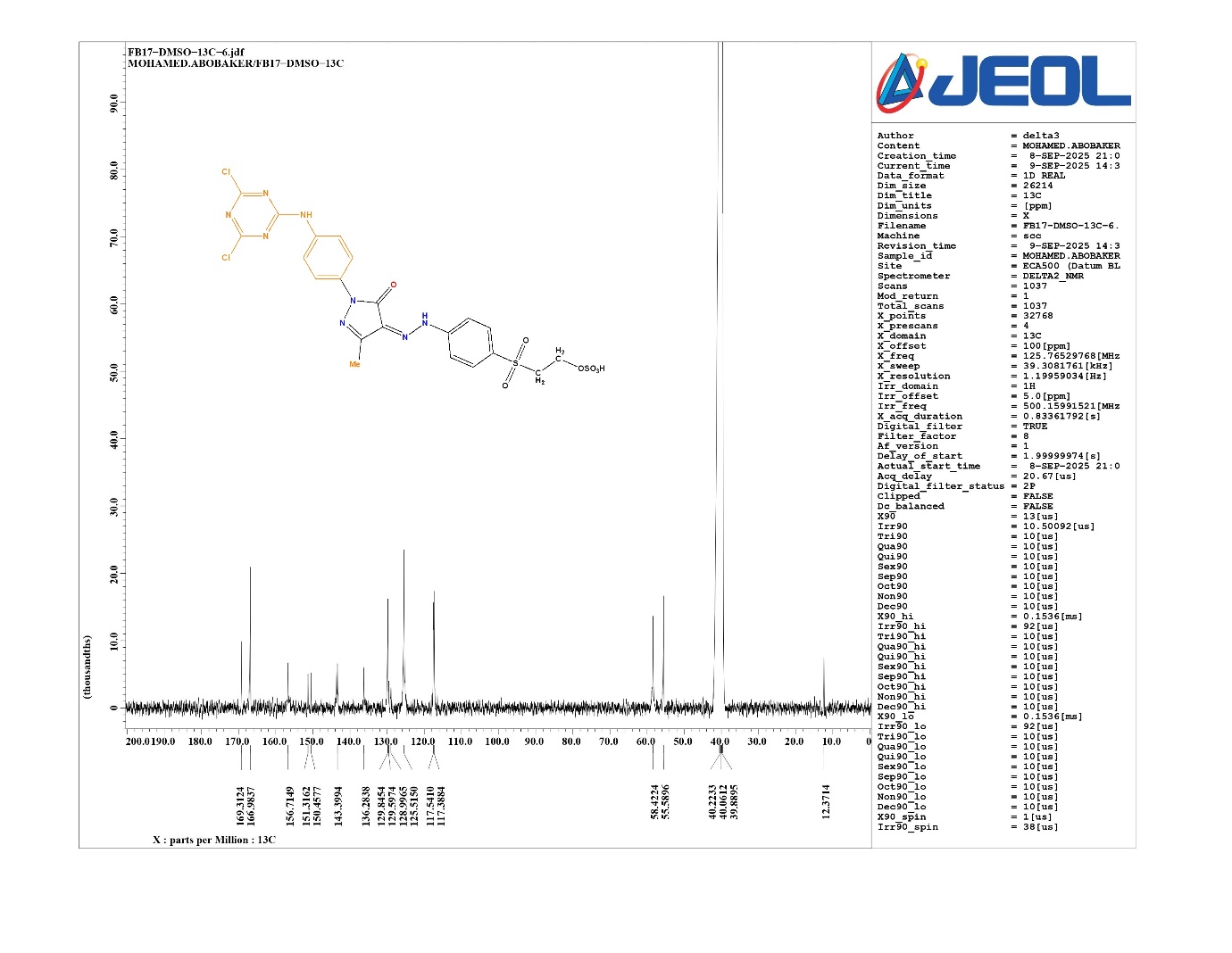


**IR spectra of the synthesized compounds 3a, 3b, and 4**


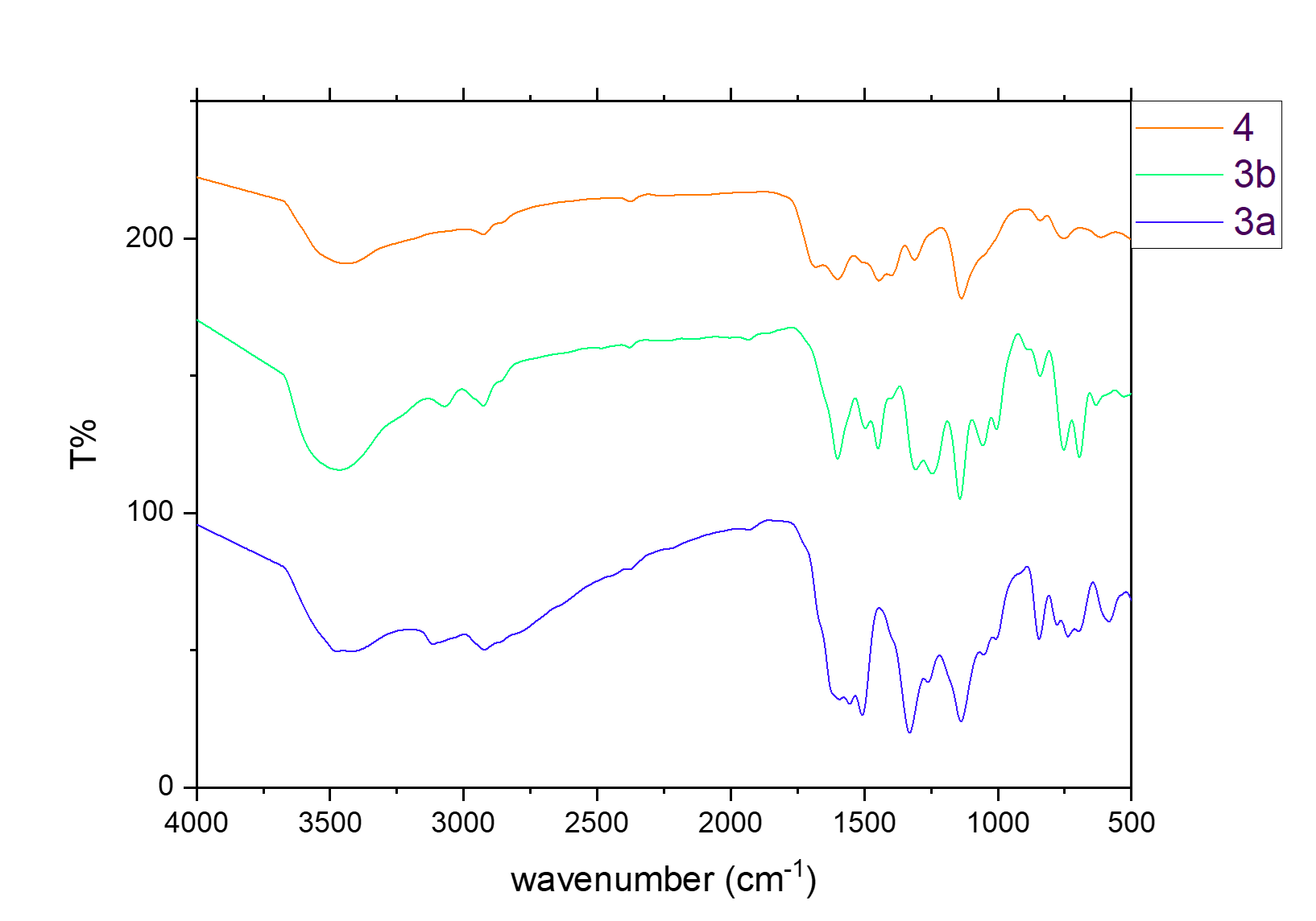


**XRD of synthesized compounds**


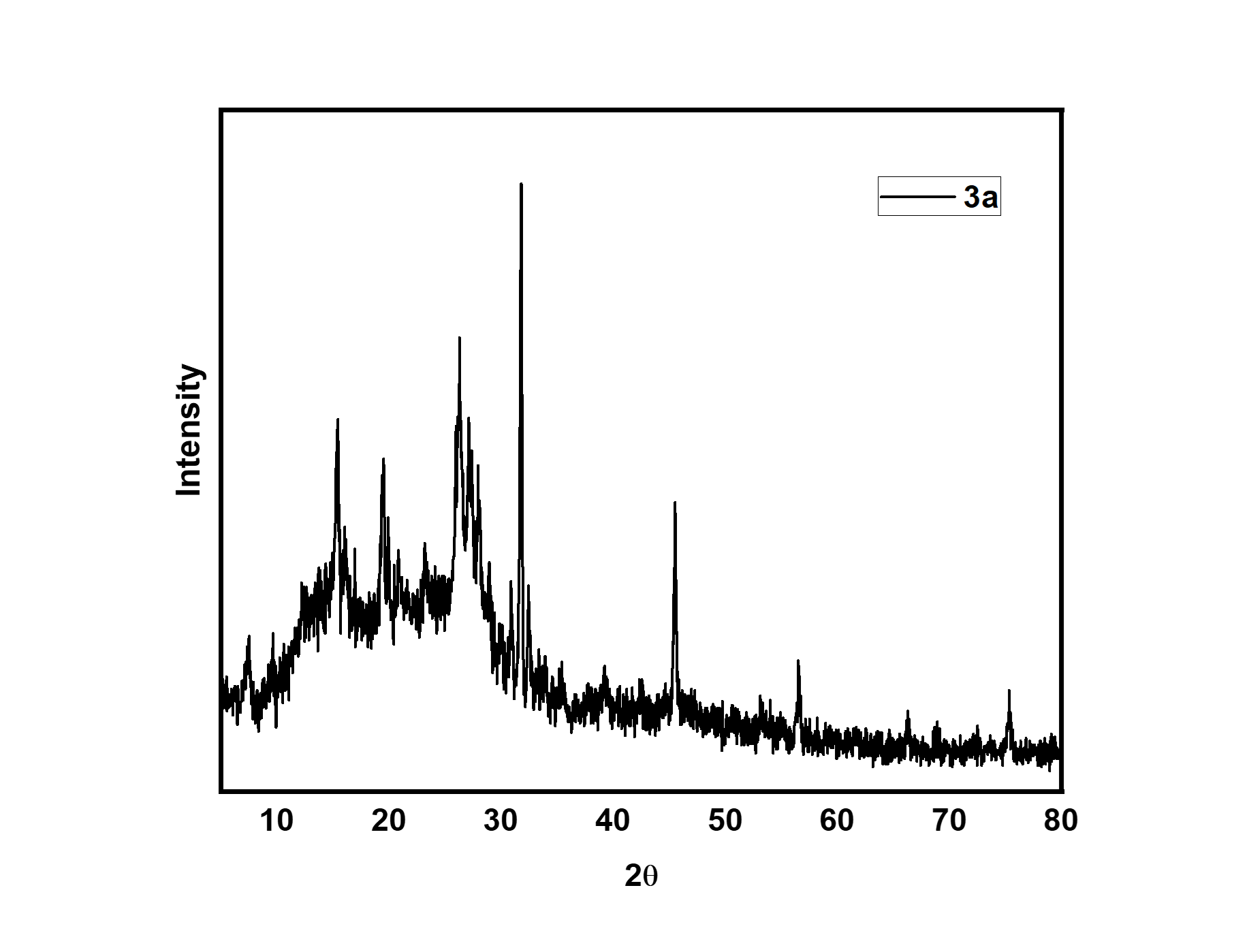


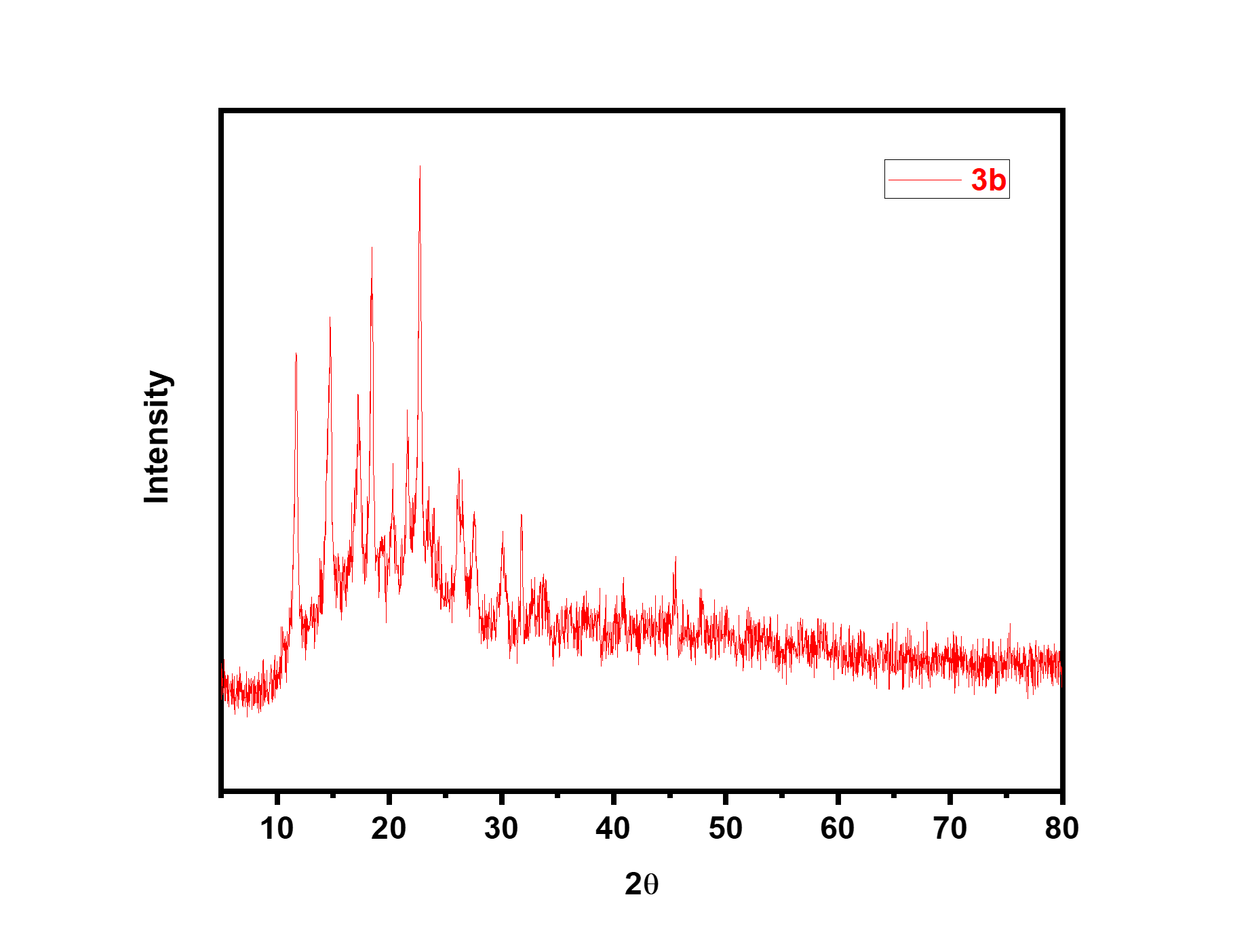

Supplement: Supplementary file 4 — Supplementary Material 4 [file 41598_2026_55597_MOESM4_ESM.docx]
